# Efficacy and safety of moxibustion treatment for upper extremity pain disorder and motor impairment in patients with stage I post-stroke shoulder-hand syndrome: a systematic review and meta-analysis of randomized controlled trials

**DOI:** 10.3389/fneur.2025.1530069

**Published:** 2025-05-23

**Authors:** Xiaonan Meng, Jie Sun, Xintong Su, David Jung Seto, Liping Wang, Ying Li, Haikuo Yu, Baixiao Zhao, Jiping Zhao

**Affiliations:** ^1^Department of Acupuncture and Moxibustion, Dongzhimen Hospital, Beijing University of Chinese Medicine, Beijing, China; ^2^Department of Acupuncture and Moxibustion, Beijing Huguosi TCM Hospital, Affiliated with Beijing University of Chinese Medicine, Beijing, China; ^3^Department of Integrated Chinese and Western Medicine Rehabilitation, Beijing Xiaotangshan Hospital, Beijing, China; ^4^Department of Acupuncture and Moxibustion, Guang’anmen Hospital, China Academy of Chinese Medical Sciences, Beijing, China; ^5^Division of Integrative Medicine, Department of Medicine, Veterans Affairs Greater Los Angeles Healthcare System, Los Angeles, CA, United States; ^6^Department of Medicine, David Geffen School of Medicine, University of California, Los Angeles, Los Angeles, CA, United States; ^7^Department of Rehabilitation, Xuanwu Hospital Capital Medical University, Beijing, China; ^8^School of Acupuncture-Moxibustion and Tuina, Beijing University of Chinese Medicine, Beijing, China

**Keywords:** moxibustion, post-stroke, upper limb, pain, shoulder-hand syndrome, systematic review, meta-analysis

## Abstract

**Background:**

Upper extremity pain disorder and motor impairment (UE-PDMI) in patients with stage I post-stroke shoulder-hand syndrome (SHS) is a common neurological comorbidity. Current interventions are with effect limitations or side effects. Moxibustion is utilized as an integrative treatment for UE-PDMI. A novel meta-analysis should be performed due to the increasing number of relevant randomized controlled trials published recently. This study aims to evaluate the efficacy and safety of moxibustion treatment for UE-PDMI.

**Methods:**

Eight databases, including the Cochrane Library, Embase, PubMed, Web of Science, China National Knowledge Infrastructure (CNKI), SinoMed database, China Science and Technology Journal Database (VIP) and WanFang database, were systematically searched, from their inception through May 15 2024, to identify potentially relevant randomized controlled trials (RCTs) on moxibustion for UE-PDMI in SHS patients. The data from the eligible RCTs was extracted by two independent investigators. The RevMan software (version 5.4.1) was employed for conducting the meta-analysis. The online GRADEpro tool was applied for rating the quality of evidence.

**Results:**

A total of 32 RCTs, involving 2,814 patients with UE-PDMI, were included. The favorable results were considered to be reflected by reduced scores on a visual analog scale (VAS) (mean difference [MD] = −1.68, 95% CI − 2.08, −1.28, *p* < 0.05), improved scores on the Fugl-Meyer Assessment of the Upper Extremity (FMA-UE, MD = 8.76, 95% CI: 7.00, 10.53, *p* < 0.05), higher scores on the modified Barthel index (MBI, MD = 10.27, 95% CI: 6.16, 14.34, *p* < 0.05) or Barthel index (BI, MD = 8.06, 95% CI: 6.20, 9.91, *p* < 0.05), and lower scores for functional impairment on National Institute of Health Stroke Scale (NIHSS, MD = −2.34, 95% CI: −2.96, −1.72, *p* < 0.05) when moxibustion was combined with rehabilitation training (RT), in contrast to control groups that implemented RT alone. The better total effective rates (TERs) were achieved when moxibustion was combined with RT (risk ratio [RR] = 1.27, 95% confidence interval [CI]:1.21, 1.33, *p* < 0.05) or with western medicine (RR = 1.18, 95% CI: 1.02, 1.35, *p* = 0.02) in comparisons to corresponding control groups. There was no significant difference in the occurrence of adverse events (AEs) between corresponding experimental and control groups (RR = 1.62, 95% CI: 0.63, 4.16, *p* > 0.05).

**Conclusion:**

This study demonstrates that moxibustion as an adjuvant therapy may play a positive role in relieving pain and improving upper extremity motor function for patients with stage I SHS, given its convenience in generating prolonged effects in communities. However, a larger number of rigorously designed, pre-registered RCTs are highly needed to verify its clinical efficacy with a higher level of certainty.

**Systematic review registration:**

https://www.crd.york.ac.uk/prospero/, identifier [CRD42024601605].

## Introduction

Stroke, ranked as the third leading contributor to global mortality and disability, imposes substantial socioeconomic burdens worldwide (1, 2). Of particular concern, approximately 50% of stroke survivors develop into shoulder-hand syndrome (SHS), with 50–60% progressing to chronic upper extremity pain disorder and motor impairment (UE-PDMI) that persists as long-term sequelae (3–5). Stage I SHS, clinically equivalent to complex regional pain syndrome type I (CRPS-I), presents with characteristic symptoms including localized hyperalgesia, dystonic spasticity, and functional deficits in motor coordination/dexterity (6, 7). This condition poses significant clinical challenges in halting its progression from stage I to terminal stages marked by irreversible muscular atrophy and joint dysfunction (8). Current therapeutic strategies for UE-PDMI in SHS encompass non-pharmacological and pharmacological interventions (9, 10). Rehabilitation training (RT), while remaining a cornerstone non-pharmacological approach with recent technological advancements, carries an inherent limitation: the induction of procedure-related pain that frequently compromises treatment adherence (11, 12). Emerging adjunctive therapies such as robot-assisted rehabilitation and virtual reality-based programs, though mechanistically promising, face implementation barriers due to prohibitive costs and unresolved technical constraints (13). Neurostimulation, encompassing both invasive and non-invasive neuromodulation techniques, has gained traction as an alternative UE-PDMI intervention (9). Contemporary high-grade randomized controlled trials (RCTs) substantiate that neurostimulation-enhanced RT protocols yield superior functional recovery outcomes (14–16). Nevertheless, invasive modalities carry inherent risks of adverse events and cost inefficiency (17, 18). Adjunctive pharmaceutical treatments involve employing a variety of western medications (WMs) for the alleviation of pain and spasticity resulting from stroke-induced hemiplegia. Non-steroidal anti-inflammatory drugs (NSAIDs) are prescribed for pain relief and muscle relaxants are utilized to decrease muscle hypertonicity, whereas the effects of these treatments are temporary, and with the potential side effect (7). Therefore, additional research is urgently needed to examine other safe, efficacious and affordable therapeutic options for UE-PDMI due to the lack of satisfactory solutions.

Moxibustion is an essential component of traditional Chinese Medicine (TCM) that has been widely utilized for over 2,500 years for both disease prevention and treatment (19). Various types of moxibustion treatment exist, including direct and indirect approaches (19). Within the indirect moxibustion category, additional subcategories also exist, such as suspended moxibustion (SM), ginger-partitioned moxibustion (GPM), herbal-partitioned moxibustion (HPM), heat-sensitive moxibustion (HSM) and warm needling moxibustion (WNM), which, respectively, involve ignited moxa floss being suspended over acupoints at a short distance, with the use of insulating materials such as air, ginger slices, herbal preparations, and acupuncture needles. The direct moxibustion category also includes subcategories such as thunder-fire moxibustion (TFM), herbal thread moxibustion (HTM) and wheat-grain moxibustion (WGM), in which ignited moxa floss in the shape of sticks, threads or cones is placed in direct contact with acupoints and then removed. Detailed information regarding these techniques is provided in Supplementary Table 1. Moxibustion is administered for a wide range of indications, owing to its clinical effect, convenience and cost-effectiveness (20). Most recently, it has even become endorsed in guidelines by a major medical society that now recommends its use for the treatment of cancer-related fatigue (21). In the setting of UE-PDMI, moxibustion appears to be effective for both alleviating pain and improving motor function, wisth its underlying mechanism of action potentially being related to its ability to induce capillary expansion, activate blood and lymphatic circulation, alleviate circulatory congestion and induce analgesia through thermal effects, infrared radiation and the biochemical actions of its chemical constituents (19, 22).

In the course of our literature search, we have found only one existing systematic review (SR) published in a Chinese medical journal in 2021 evaluated the efficacy of moxibustion for the treatment of post-stroke SHS (23). From a methodological perspective, this study exhibits the following critical limitations. The literature search strategies for both Chinese and English databases were insufficiently detailed, potentially missing relevant studies, which raises concerns about the comprehensiveness of evidence retrieval and possible publication bias. Also, the methodology failed to explicitly state adherence to PRISMA (Preferred Reporting Items for Systematic Reviews and Meta-Analyses) guidelines. For instance, the PRISMA flowchart lacks specific details on the number of excluded studies and reasons for exclusion (e.g., vague criteria such as “non-randomized controlled trials” without clarifying screening procedures). Meanwhile, no adverse events (e.g., burns, allergic reactions) were reported across the included studies, potentially underestimating treatment risks and compromising safety assessments. Moreover, the sensitivity analysis merely described the removal of high-heterogeneity studies without quantitatively detailing effect size changes (e.g., omitting numerical comparisons of MD [mean difference] or CI [confidence interval] pre- and post-exclusion). Furthermore, the sources of heterogeneity (e.g., variations in intervention protocols, patient demographics) were not rigorously investigated through subgroup analyses or meta-regression. Poor methodological transparency (e.g., ambiguous exclusion criteria, incomplete reporting of search strategies) and unresolved heterogeneity compromised the credibility of the findings. Given that additional relevant studies have been newly published since this review was performed, there is a need for researchers to conduct a novel SR and meta-analysis with more rigorous methodological design in order to update the global understanding of the safety and clinical efficacy of moxibustion in the context of UE-PDMI in stage I SHS patients.

## Materials and methods

All data was extracted from previously published studies, thus neither Institutional Review Board (IRB) approval nor patient consent was required to be obtained for this analysis. This study was conducted in accordance with the Preferred Reporting Items for Systematic Reviews and Meta-Analysis (PRISMA) 2020 statement (shown in Supplementary Table 2) (24), and was pre-registered in the International Prospective Register of Systematic Reviews (PROSPERO) with the registration identifier number CRD42024601605.

### Search strategy and study selection

Eight databases, including the Cochrane Library, PubMed, Embase, Web of Science, the China National Knowledge Infrastructure (CNKI) database, the China Science and Technology Journal Database (VIP), the SinoMed database and the WanFang database, were systematically searched for relevant RCTs, from their inception to May 15, 2024. The detailed database search methodology is presented in the Supplementary material section, in accordance with Population, Intervention, Comparison, Outcomes, and Study Design (PICOS) criteria. Initially identified articles were imported into the NoteExpress software, and their titles and abstracts were screened in order to eliminate duplicate records. Subsequently, a thorough full-text review of potentially eligible studies was conducted. Data from eligible RCTs was extracted and filtered meticulously by two independent investigators to maintain selection integrity. Any disagreements regarding study eligibility were resolved through arbitration with a third independent investigator.

### Inclusion criteria

Eligible studies for inclusion met the following criteria: (1) Population: Patients diagnosed with cerebral stroke confirmed by computed tomography (CT) or magnetic resonance imaging (MRI) (25), and experiencing pain and motor impairment in the affected upper extremity due to stage I SHS (7, 26), regardless of gender, ethnicity or any other demographic factors. (2) Regarding moxibustion in the experimental groups, SM, HSM, GPM, HPM, WNM, TFM and WGM were included without any further subtype limitations. RCTs were included if moxibustion in experimental groups was utilized as monotherapy or as an adjuvant therapy to the same interventions that were utilized in the control groups. (3) Comparison: Interventions in the control groups were limited to WMs or RT in order to evaluate the true effectiveness of moxibustion for UE-PDMI. RCTs that compared moxibustion against other TCM therapies, or that compared different moxibustion techniques against each other, were not included. (4) Outcomes: The primary outcomes included a visual analog scale (VAS) for pain and the Fugl-Meyer Assessment of the Upper Extremity (FMA-UE) scale. The secondary outcomes included the total effective rate (TER) of recovery, the Modified Barthel Index (MBI), Barthel Index (BI), National Institute of Health Stroke Scale (NIHSS) and the reporting of adverse events (AEs). (5) Study Design: Only RCTs were eligible, with no restrictions on the language of publication.

### Data collection process and study quality assessment

All data extracted from the eligible trials was compiled using Microsoft Excel software, including general characteristics, demographics, types of interventions, outcome indicators and AEs. The responsible investigators meticulously cross-checked all data to ensure its accuracy. For RCTs with multiple-arm designs, irrelevant data from other experimental arms was not analyzed. We reached out to the corresponding authors whenever there appeared to be missing information or potential ambiguities that could not be directly clarified from the articles themselves. The Cochrane Risk of Bias (ROB) 1.0 tool was utilized to assess each publication for the risk of bias, with each of seven domains being assessed and rated as conveying a low, high or unclear level of risk for bias (27). Moreover, given that numerous moxibustion studies were reported in Chinese journals, the Consolidated Standards for Reporting of Trials (CONSORT) statement was also applied to evaluate the quality of the included RCTs quality (28). The reporting percentages for each item in the CONSORT statement are listed the Supplementary Table 3.

### Data synthesis and statistical methods

The Cochrane ReviewManager software (version 5.4.1) was utilized for conducting the meta-analysis, with Chi-squared tests (*p*-value) and I^2^ values (percentage) being used for statistical evaluation and the quantification of heterogeneity among studies, respectively. A random effect (RE) model was selected with significant heterogeneity (*p* < 0.1, I^2^ > 50%), whereas a fixed effect (FE) model was chosen for studies with acceptable levels of heterogeneity (*p* ≥ 0.1, I^2^ ≤ 50%). Dichotomous variables were assessed using risk ratio (RR) with 95% confidence intervals (CI), whereas continuous variables were assessed by mean difference (MD) or standardized mean difference (SMD) with 95% CI. The statistically significant differences of the pooled outcomes (overall effects) were calculated using Z-test with *p* < 0.05 (two-sided). Our team categorized included studies according to the types of control groups that they implemented, i.e., RT vs. WM. Meanwhile, subgroup analysis was performed to mitigate any possible heterogeneity that was introduced via the stratified factor of various forms of moxibustion that were implemented in different trials. The robustness of the results of this meta-analysis was verified through sensitivity analysis, which aimed to evaluate the effects of possible sources of heterogeneity. Publication biases for the outcomes from over ten of the included trials were assessed and demonstrated using funnel plots (29). The online GRADEpro Guideline Development Tool (GDT) was utilized to rate the quality of evidence and the level of certainty regarding therapeutic effects that were observed (30).

## Results

### Literature search and study characteristics

**Figure 1:**
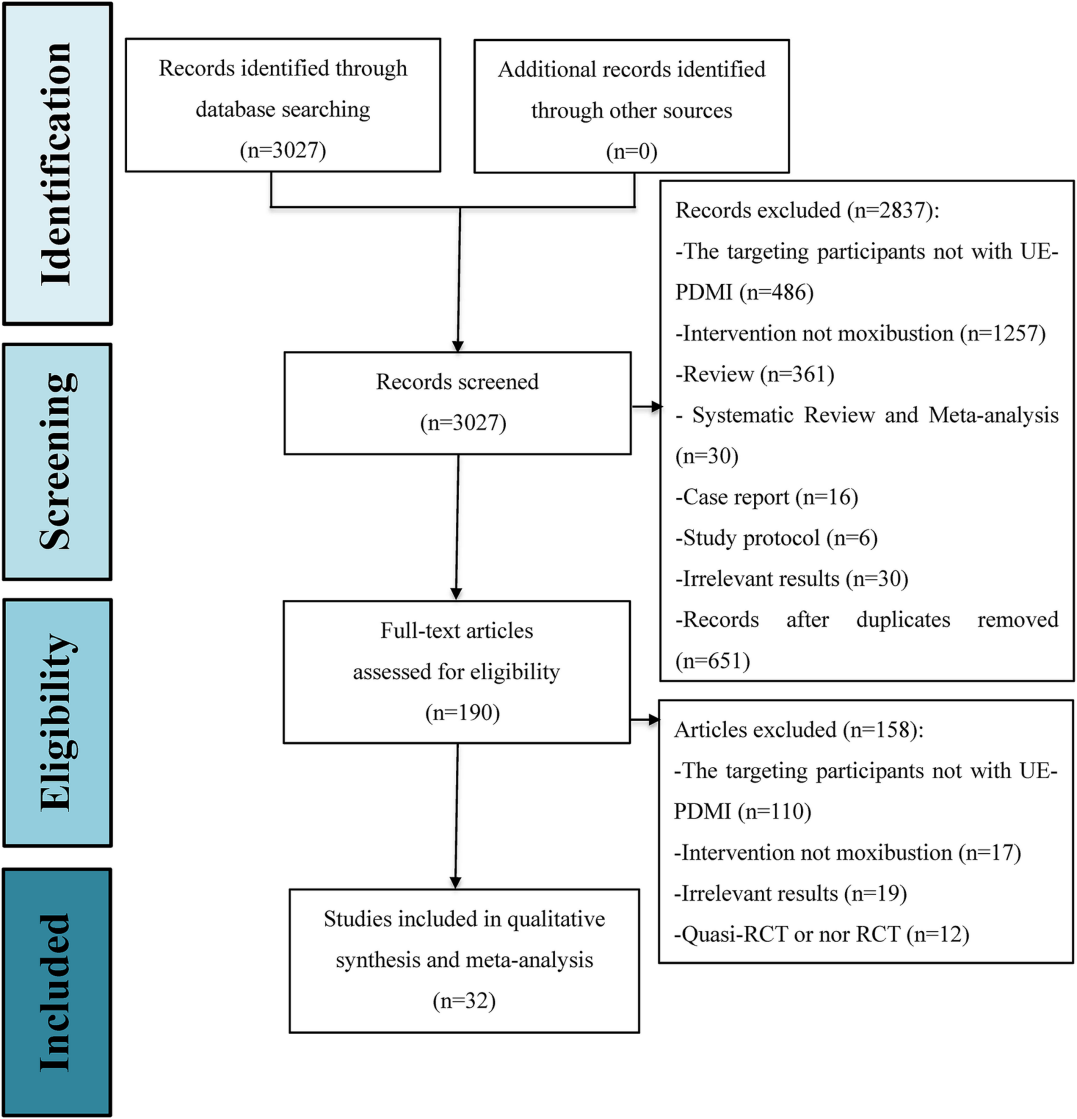
Inclusion process flowchart.

A total of 3,027 citations from the eight databases were identified through an initially comprehensive search. Following screening to eliminate duplicate records and a close reading of titles and abstracts, the full texts of 190 potential studies were reviewed in detail. Ultimately, 32 eligible RCTs, with a total of 2,814 stage I SHS patients suffering from UE-PDMI were included for examination in this meta-analysis, all of which were conducted in China, with three articles published in English (31–63). The PRISMA flowchart for illustrating the inclusion process is demonstrated in Figure 1. These studies included patients who had suffered from either ischemic or hemorrhagic types of strokes, with the proportion of ischemic strokes accounting for 75.7% of the cases. All 32 trials employed a two-group parallel comparison design with 57.7% of the patients being male, and with a total of 1,412 patients being assigned to control groups and 1,402 patients being assigned to treatment groups. Among all of the comparison pairs, only two studies compared moxibustion against WM alone (41, 48), while the rest of the trials compared moxibustion plus RT against RT alone. Sample sizes ranged from n = 56 up to n = 128. Eleven studies (34.4%) were funded by scientific research grants from the Chinese government, whereas the other 21 studies (65.6%) did not mention any funding sources. Detailed information for all of the included studies is shown in Table 1.

**Table 1:** Characteristics of included studies.

Included studies	Sample size (T, C)	Age (y) (Mean) (T, C)	Gender (Male/Female) (T, C)	Stroke type (I/H) (T, C)	Stroke duration (T, C)	Moxibustion					Control type	Outcome indicators	Assessment time point
						Style	Course (w)	Frequency (t/w)	Intervention (min)	Regional sensation			
Chen et al. 2023 (60)	35/35	52.91, 53.69	18/17, 19/16	NR	3.08 m, 3.19 m	SM + RT	3	5	20	Mildly warm	RT	TER, FMA-UE, BI	3w
Tong et al. 2023 (62)	30/29	60.47, 59.94	15/15, 14/15	23/7, 21/8	78.54d, 80.33d	GPM + RT	6	3	NR	Mildly warm	RT	TER, FMA-UE, MBI	6w
Gao et al. 2023 (61)	48/49	63, 64	37/11, 40/9	34/14, 36/13	2.26 m, 2.57 m	WGM + RT	4	5	NR	Mildly burning	RT	TER, FMA-UE, BI, VAS	4w
Wang et al. 2023 (63)	30/30	64.17, 65.50	15/15, 14/16	22/8, 20/10	90.67d, 88.00d	SM + RT	4	6	NR	Mildly warm	RT	TER, FMA-UE, MBI	4w
Huang 2022 (57)	30/30	60.22, 60.13	16/14, 17/13	NR	3.88w, 3.79w	SM + RT	4	5	NR	Mildly warm	RT	FMA-UE, BI	4w
Ding et al. 2022 (56)	45/45	58.12, 57.32	24/21, 21/24	25/20, 27/18	50.10d, 48.39d	GPM + RT	6	1	60	NR	RT	FMA-UE, BI	6w
Niu et al. 2022 (58)	40/40	53, 55	21/19, 25/15	NR	3.6 m, 3.4 m	WGM + RT	4	6	NR	Mildly burning	RT	TER, MBI	4w
Sun et al. 2022 (59)	28/28	57.71, 63.00	17/11, 16/12	22/6, 21/7	1.86 m, 1.78 m	SM + RT	6	5	30	Mildly warm	RT	FMA-UE, MBI	6w
Zhao et al. 2022 (55)	30/30	56.5, 60.2	19/11, 16/14	20/10, 21/9	2.7 m, 2.7 m	SM + RT	4	5	20	Mildly warm	RT	VAS, FMA-UE, BI, NIHSS	4w
Huang 2021 (52)	44/44	63.54, 63.56	25/19, 24.20	38/6, 37,7	3.95 m, 3.93 m	WNM + RT	8	5	NR	Mildly warm	RT	FMA-UE	8w
Xie et al. 2021 (54)	53/53	59.64, 60.01	28/25, 24/29	32/21, 32/21	29.23d, 30.18d	WNM + RT	12	5	NR	Mildly warm	RT	TER, VAS, FMA-UE	12w
Wei et al. 2021 (53)	51/51	58, 57	25/26, 28/23	NR	23d, 23d	WNM + RT	4	5	30	Mildly warm	RT	TER, FMA-UE	4w
Yu-chun 2021 (51)	50/50	67.6, 66.8	28/22, 26/24	50/0, 50,0	3.9 m, 3.8 m	GPM + RT	4	1	NR	NR	RT	TER, FMA-UE, BI	4w
Zhang et al. 2020 (50)	60/60	46.79, 46.27	35/25, 33/27	NR	25.55d, 25.72d	HSM + RT	12	5	40	Mildly warm	RT	TER, VAS, FMA-UE, BI	12w
Liu et al. 2020 (49)	40/40	61.7, 62.3	25/15, 27/13	27/13, 29/11	NR	HSM + RT	4	5	60	Mildly warm	RT	FMA-UE, MBI	4w
Li et al. 2020 (48)	40/40	54.21, 53.87	22/18, 23/17	NR	61.25d, 62.31d	WNM	5	6	NR	Mildly warm	WM	TER, MAS, FMA-UE, BI	5w
Hu 2019 (47)	43/43	68.4, 67.8	20/23, 22/21	28/15, 30/13	18.4d, 18.2d	HPM + RT	4	5	20	Mildly warm	RT	TER, VAS, FMA-UE	4w
Gu 2019 (46)	41/41	58.23, 60.46	21/20, 23/18	NR	23.51d, 24.78d	WNM + RT	4	6	NR	Mildly warm	RT	TER, MBI, VAS	4w
Chen et al. 2017 (41)	33/33	55.28, 57.03	20/13, 22/11	NR	39.01d, 37.94d	HPM	2.1	7	NR	Mildly warm	WM	TER, VAS, MBI	15d
Wang et al. 2017 (45)	56/55	61.4, 60.8	36/20, 34/21	NR	40.7d, 39.4d	WNM + RT	3	6	25	Mildly warm	RT	FMA-UE, NIHSS	3w
Huang et al. 2017 (42)	56/48	62.8, 59.7	25/31, 20/28	56/0, 48/0	NR	HSM + RT	NR	NR	NR	Mildly warm	RT	TER, FMA-UE	NR
Liu 2017 (44)	30/30	NR	NR	NR	NR	WNM + RT	4	5	20	Mildly warm	RT	TER, FMA-UE	4w
Chen et al. 2016 (40)	40/40	57.2, 55.5	22/18, 24/16	NR	NR	HSM + RT	4	5	40	Mildly warm	RT	TER, VAS, FMA-UE	4w
Xu 2015 (39)	52/52	49.2, 51.3	28/24, 26/26	NR	31.6d, 31.9d	HTM + RT	3	7	NR	NR	RT	VAS, FMA-UE	3w
Zhao et al. 2015 (38)	62/62	63.73, 65.53	44/18, 45/17	51/11, 53/9	120.0d, 104.5d	WNM + RT	2	5	40	Mildly warm	RT	VAS, BI	2w
Chang et al. 2013 (34)	52/52	NR	39/13, 37/15	33/19, 32/20	NR	TFM + RT	6	5	15	Mildly warm	RT	TER	6w
Jin et al. 2013 (36)	30/30	61.48, 60.51	16/14, 17/13	23/7, 22/8	NR	HSM + RT	4	7	40	Mildly warm	RT	TER, FMA-UE	4w
He et al. 2013 (35)	60/61	65.25, 64.70	37/23, 39/22	NR	NR	HTM + RT	4	6	NR	Mildly warm	RT	TER, FMA-UE, BI	4w
Shen et al. 2013 (37)	40/40	63.57, 65.21	22/18, 16/24	27/13, 32/8	NR	WNM + RT	4	NR	30	Mildly warm	RT	FMA-UE, MBI	4w
Du et al. 2012 (33)	38/38	NR	23/15, 25/13	29/9, 31/7	NR	HPM + RT	3	7	20	Mildly warm	RT	VAS, FMA-UE	3w
Qiu 2011 (32)	60/60	NR	NR	NR	NR	WNM + RT	8	5	NR	Mildly warm	RT	VAS, FMA-UE, MBI	8w
Han 2010 (31)	65/63	54.32, 53.64	41/24, 38/25	NR	3.32 m, 3.45 m	WNM + RT	4	5	30	Mildly warm	RT	TER, FMA-UE	4w

RCT, randomized controlled trial; RT, rehabilitation training; SM, suspended moxibustion; GPM, ginger-partitioned moxibustion; WGM, wheat-grain moxibustion; WNM, warm needling moxibustion; HSM, heat-sensitive moxibustion; HPM, herb-partitioned moxibustion; TFM, thunder-fire moxibustion; HTM, herbal thread moxibustion; NR, not reported; TER, total effective rate; FMA-UE, Fugl-Meyer Assessment of the Upper Extremity; MBI, modified Barthel Index; BI, Barthel Index; VAS, visual analog scale; NIHSS, National Institute of Health Stroke Scale.

As mentioned in the introduction section, eight different types of moxibustion were represented in the included RCTs, including both direct and indirect methods. WNM (34.4%) was the most commonly employed moxibustion type, followed by SM (15.6%) and HSM (15.6%). The duration of treatment courses ranged from 2 to 12 weeks with a considerable variation. Nevertheless, longer therapeutic courses for no less than 4 weeks were preferred in more than 78.1% of RCTs (n = 25). The frequency ranged from 1 to 7 sessions per week, with the majority involving high-frequency treatments in the range of 5–7 sessions per week (n = 27). The duration and dosing of each moxibustion session ranged from 15–60 min or a minimum of 3 cones. Notably, all studies emphasized the experience of regional sensation during moxibustion. Two studies utilizing WGM (56, 61), (a form of direct moxibustion that involves placing wheat grain-sized moxa cones measuring 3 millimeters in diameter and 3–4 millimeters in height on the skin and igniting them without insulation), noted subjects’ report of regional sensation to be “mildly burning,” whereas the other 30 studies, which involved a variety of moxibustion types, all noted subjects’ report of regional sensation to be “mildly warming.” No severe adverse events were reported in any of the studies, even including those that involved direct moxibustion. The top ten most frequently applied acupoints were LI 4 (43.8%), LI 11 (43.8%), LI 14 (43.8%), LI 15 (40.6%), TE 5 (40.6%), LI 10 (37.5%), TE 14 (34.4%), SI 9 (18.8%), PC 6 (12.5%), and LU 5 (12.5%). Acupoint combinations generally involved regional points located on and around the affected extremity, although distal acupoints were also utilized as well. Detailed information about acupoint selection is summarized in Table 2.

**Table 2:** Acupoint selection.

Included studies	Style	Major acupoint combination	Meridian attribution(s)
Chen et al. 2023 (60)	SM	Shen-zhu (GV 12), Ling-tai (GV 10), Zhi-yang (GV 9), Tao-dao (GV 13), Zhong-shu (GV 7), Shen-dao (GV 11), Xuan-shu (GV 5), Jin-suo (GV 8), Yao-yang-guan (GV 3), Ji-zhong (GV 6), Ming-men (GV 4)	GV
Tong et al. 2023 (62)	GPM	Da-zhui (GV14), Tao-dao (GV 13), Shen-zhu (GV 12), Shen-dao (GV 11), Ling-tai (GV 10), Zhi-yang (GV 9), Jin-suo (GV 8), Zhong-shu (GV 7), Ji-zhong (GV 6), Xuan-shu (GV 5), Ming-men (GV 4), Yao-yang-guan (GV 3), Yao-shu (GV 2)	GV
Gao et al. 2023 (61)	WGM	Jian-yu (LI 15), Bi-nao (LI 14), Jian-liao (TE 14), Jian-zhen (SI 9), Qu-chi (LI 11), Zu-san-li (ST 36) (affected side)	LI, TE, SI, ST
Wang et al. 2023 (63)	SM	Bai-hui (GV 20), Shen-que (CV 8)	GV, CV
Huang 2022 (57)	SM	He-gu (LI 4), Zhong-wan (CV 12), Wai-guan (TE 5) (affected side)	LI, CV, TE
Ding et al. 2022 (56)	GPM	Da-zhui (GV14), Tao-dao (GV 13), Shen-zhu (GV 12), Shen-dao (GV 11), Ling-tai (GV 10), Zhi-yang (GV 9), Jin-suo (GV 8), Zhong-shu (GV 7), Ji-zhong (GV 6), Xuan-shu (GV 5), Ming-men (GV 4), Yao-yang-guan (GV 3), Yao-shu (GV 2)	GV
Niu et al. 2022 (58)	WGM	Shi-xuan (EX-UE 11)	EX
Sun et al. 2022 (59)	SM	A-shi acupoint	EX
Zhao et al. 2022 (55)	SM	Zhou-liao (LI 12), Jian-yu (LI 15), Nei-guan (PC 6), Zhi-gou (TE 6), Jian-zhen (SI 9), Shou-san-li (LI 10)	LI, PC, SI, TE
Huang 2021 (52)	WNM	Jian-yu (LI 15), Bi-nao (LI 14), Jian-liao (TE 14), Jian-zhen (SI 9), Ju-gu (LI 16), Bai-xie (EX-UE 9), Qi-hai (CV 6), He-gu (LI 4), Hou-xi (SI 3), Wai-guan (TE 5) (affected side)	LI, EX, TE, CV
Xie et al. 2021 (54)	WNM	Ji-quan (HT 1), Chi-ze (LU 5), Jian-liao (TE 14), Qu-chi (LI 11), Shou-san-li (LI 10), He-gu (LI 4), Wai-guan (TE 5), Nei-guan (PC 6) (affected side)	HT, LU, TE, LI, PC
Wei et al. 2021 (53)	WNM	Qu-chi (LI 11), Wai-guan (TE 5), Nei-guan (PC 6) (affected side)	LI, PC, TE
Yu-chun 2021 (51)	WNM	Da-zhui (GV14), Tao-dao (GV 13), Shen-zhu (GV 12), Shen-dao (GV 11), Ling-tai (GV 10), Zhi-yang (GV 9), Jin-suo (GV 8), Zhong-shu (GV 7), Ji-zhong (GV 6), Xuan-shu (GV 5), Ming-men (GV 4), Yao-yang-guan (GV 3), Yao-shu (GV 2)	GV
Zhang et al. 2020 (50)	HSM	A-shi acupoints	EX
Liu et al. 2020 (49)	HSM	Shou-san-li (LI 10), He-gu (LI 4), Ba-xie (EX-UE 9) (affected side)	LI, EX
Li et al. 2020 (48)	WNM	Jian-yu (LI 15), Wai-guan (TE 5), Shou-san-li (LI 10), Qu-chi (LI 11) (affected side)	LI, TE
Hu 2019 (47)	HPM	Jian-yu (LI 15), Jian-liao (TE 14), Wai-guan (TE 5), Qu-chi (LI 11), He-gu (LI 4) (affected side)	LI, TE
Gu 2019 (46)	WNM	NR	NR
Chen et al. 2017 (41)	HPM	Jian-jing (GB 21), Tian-zong (SI 11), Jian-liao (TE 14), Chi-ze (LU 5), Wai-guan (TE 5), He-gu (LI 4) (affected side)	GB, SI, TE, LU, LI
Wang et al. 2017 (45)	WNM	Jian-liao (TE 14), Tian-zong (SI 11), Qu-chi (LI 11), Wan-gu (SI 4), He-gu (LI 4), Wai-guan (TE 5), Lie-qie (LU 7), Shou-san-li (LI 10) (affected side)	TE, SI, LI, LU
Huang et al. 2017 (42)	HSM	Bai-hui (GV 20), Feng-chi (GB 20), Shou-san-li (LI 10) (affected side if appliable)	GV, GB, LI
Liu 2017 (44)	WNM	Jian-yu (LI 15), Bi-nao (LI 14), Chi-ze (LU 5), Qu-chi (LI 11), Zu-san-li (ST 36), Wai-guan (TE 5), He-gu (LI 4), Tai-yuan (LU 9), Xuan-zhong (GB 39) (affected side)	LI, LU, ST, TE, GB
Chen et al. 2016 (40)	HSM	Bai-hui (GV 20), Feng-chi (GB 20), Shou-san-li (LI 10) (affected side if appliable)	GV, GB, LI
Xu 2015 (39)	HTM	Jian-yu (LI 15), Jian-liao (TE 14), Qu-chi (LI 11), He-gu (LI 4), A-shi acupoints (affected side)	LI, TE, EX
Zhao et al. 2015 (38)	WNM	Jianyuci (sub-LI 15), Binaoci (sub-LI 14), Jianzhenci (sub-SI 9), Jianliaoci (sub-TE 14), Tianfuci (sub-LU 3), Shousanlici (sub-LI 10), Yangchici (sub-TE 4)	-
Chang et al. 2015 (34)	TFM	Dazhui (GV 14), Jian-jing (GB 21), Jian-yu (LI 15), Jian-liao (TE 14), Qu-chi (LI 11), A-shi acupoints (affected side)	GV, GB, LI, TE, EX
Jin et al. 2013 (36)	HSM	He-gu (LI 4), Yang-xi (LI 5), Pian-li (LI 6), Wen-liu (LI 7), Xia-lian (LI 8), Shang-lian (LI 9), Shou-san-li (LI 10), Qu-chi (LI 11), Zhou-liao (LI 12), Shou-wu-li (LI 13), Bi-nao (LI 14), Jian-yu (LI 15) (affected side)	LI
He et al. 2013 (35)	HTM	Jian-yu (LI 15), Qu-chi (LI 11), He-gu (LI 4), Shou-san-li (LI 10), Wai-guan (TE 5) (affected side)	LI, TE
Shen et al. 2013 (37)	WNM	Ji-quan (HT 1), Chi-ze (LU 5), Nei-guan (PC 6), Wai-guan (TE 5), He-gu (LI 4), Qu-chi (LI 11) (unaffected side)	HT, LU, PC, TE, LI
Du et al. 2013 (33)	HPM	Jian-zhen (SI 9), Bi-nao (LI 14), Yang-xi (LI 5), Zhong-zhu (TE 3), He-gu (LI 4), A-shi acupoints (affected side)	SI, LI, TE, EX
Qiu 2011 (32)	WNM	Jian-liao (TE 14), Jian-yu (LI 15), Bi-nao (LI 14), Qu-chi (LI 11), Shou-san-li (LI 10), Wai-guan (TE 5), He-gu (LI 4) (affected side)	TE, LI
Han et al. 2010 (31)	WNM	Jian-liao (TE 14), Jian-yu (LI 15), Jian-zhen (SI 9), Tian-zong (SI 11), Qu-chi (LI 11), Shou-san-li (LI 10), Wai-guan (TE 5), Yang-lao (SI 6), Zhong-zhu (TE 3) (affected side)	TE, SI, LI

SM, suspended moxibustion; GV, governor vessel; GPM, ginger-partitioned moxibustion; WGM, wheat-grain moxibustion; LI, large intestine; TE, triple energizer; SI, small intestine; ST, stomach; CV, conception vessel; WNM, warm-needling moxibustion; GB, gallbladder; EX, extraordinary; UE, upper extremity; PC, pericardium; TFM, thunder-fire moxibustion; HT, heart; LU, lung; HSM, heat-sensitive moxibustion; HPM, herb-partitioned moxibustion.

### Methodological and reporting quality

**Figure 2:**
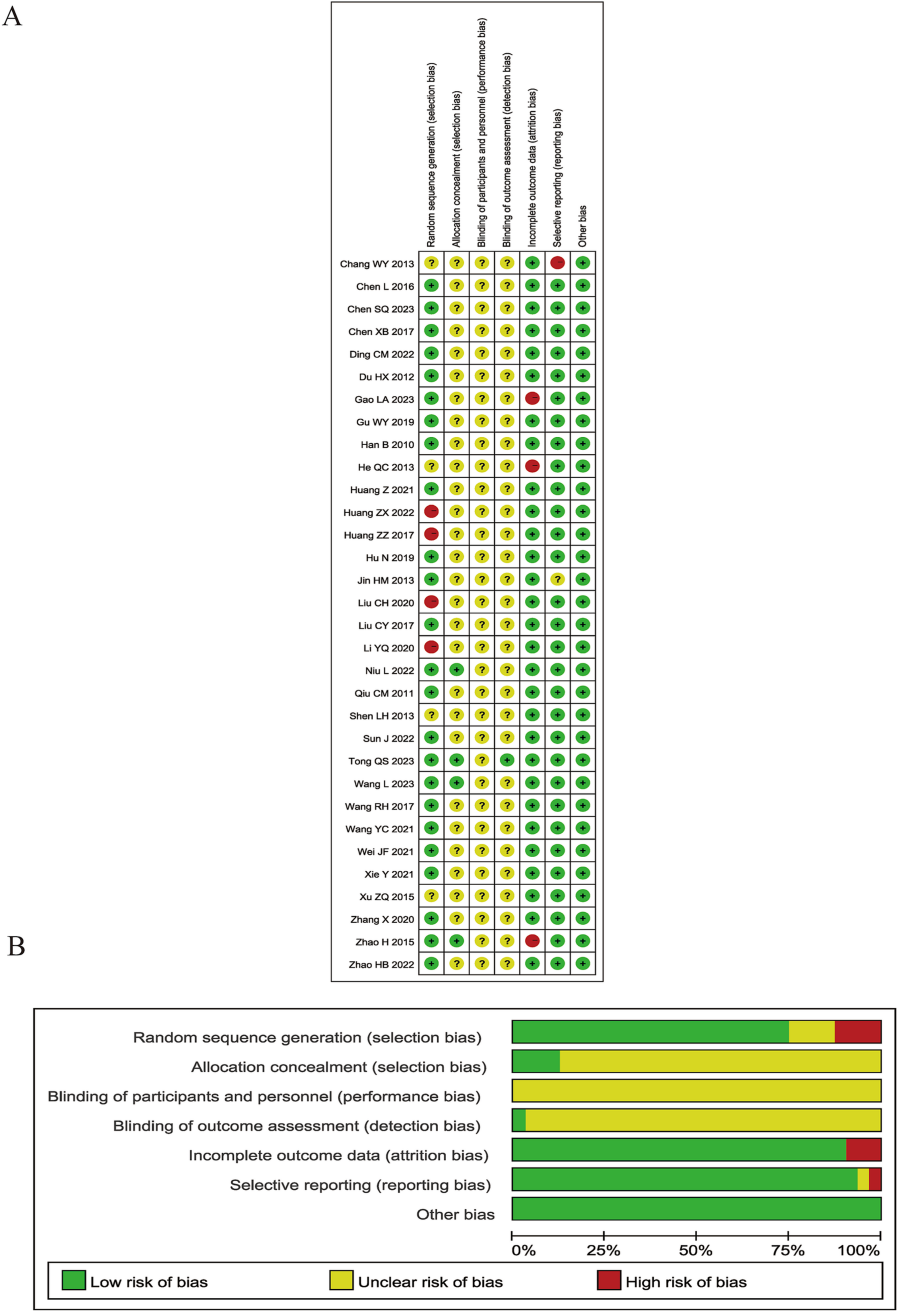
The assessment of Cochrane Risk of Bias (ROB). **(A)** ROB graph and **(B)** ROB summary.

The Cochrane Risk of Bias (ROB) 1.0 tool was applied to assess the methodological and reporting quality of all 32 RCTs included in this meta-analysis. Twenty-four RCTs (75%) were rated as “low risk of bias” through utilizing appropriate sequence generation methods such as random number tables or Statistical Package for the Social Sciences (SPSS) software. Four studies (12.5%) were rated as having “unclear risk of bias” because they only stated that “randomization” was performed, but without describing any further details about the randomization process. Four RCTs (12.5%) did not mention any measures whatsoever that were taken to ensure proper randomization, which led to them being given a rating of “high risk of bias.” Only four RCTs reported allocation concealment through the use of randomized numbers and the masking of group assignments using sealed opaque envelopes. However, no significant baseline differences existed between groups in these RCTs. None of the RCTs reported any details about blinding or sham-controlled groups, and only one trial specified the method used to blind outcome assessors. Three studies reporting drop-outs were rated as “high risk of bias” because of the absence of an intention-to-treat analysis. None of these RCTs were pre-registered with their protocols being published in advance, leading to a rating of “unclear risk of bias” in this dimension. No other bias was detected. In general, the quality of methodology and reporting in the available RCTs was not satisfactory. The results of the ROB assessment are graphically demonstrated in Figure 2 and judgment details being shown in Supplementary Table 4.

### Visual Analog Scale

**Figure 3:**
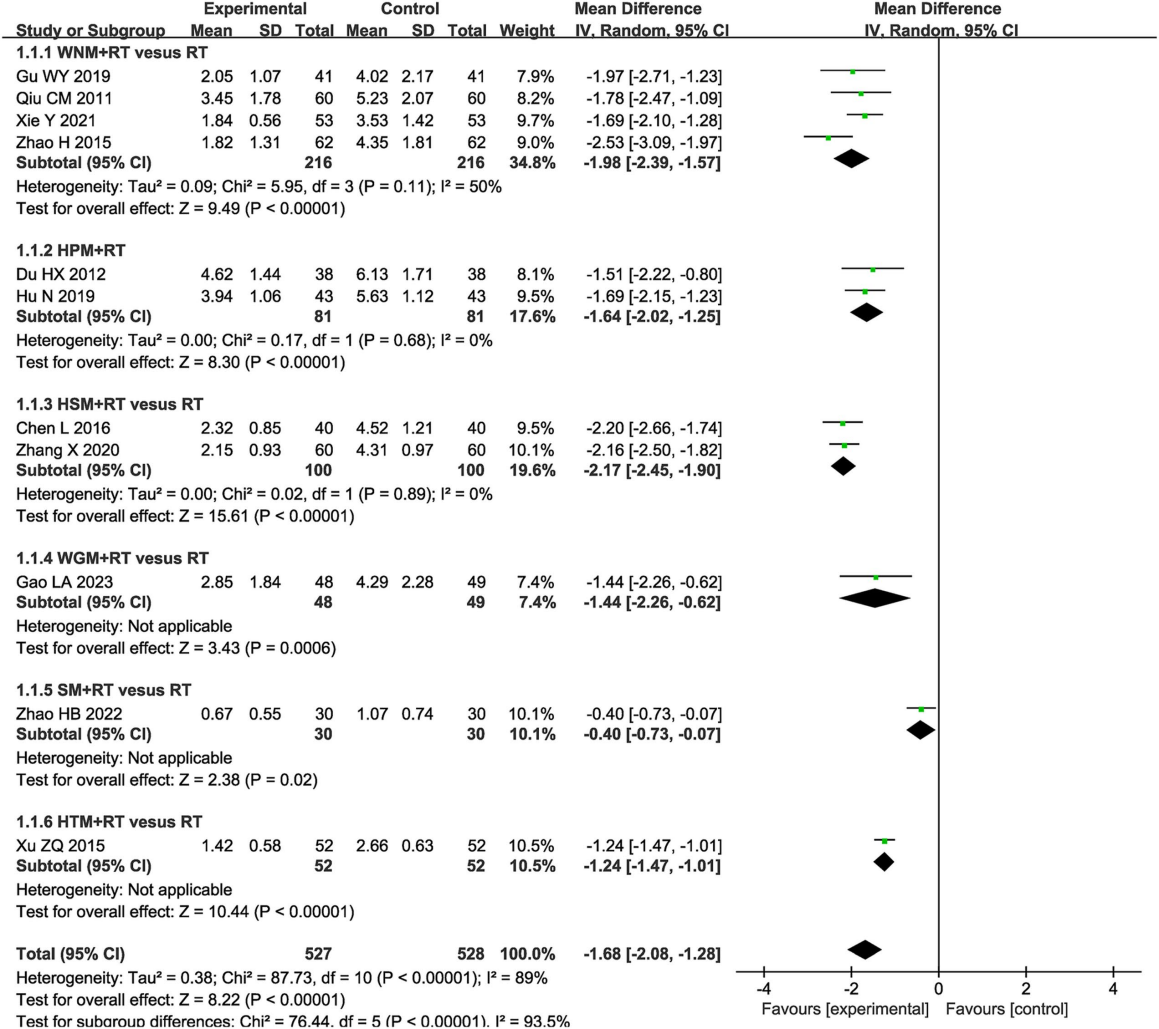
Forest plot of Visual Analog Scale (VAS) when comparing moxibustion plus RT (rehabilitation training) vs. RT alone.

A Visual Analog Scale (VAS) is the most frequently utilized tool for assessing the effect of moxibustion on pain in patients with post-stroke UE-PDMI. Eleven trials involving 1,055 patients reported VAS data with the same scale of 0–10 when comparing moxibustion plus RT against RT alone (32, 33, 38–40, 46, 47, 50, 53–55, 61). The pooled MD with a RE model revealed lower VAS scores for pain in the experimental groups employing moxibustion plus RT (MD -1.68, 95% CI -2.08– −1.28) with high heterogeneity (I^2^ = 89%, *p* < 0.00001, shown in Figure 3).

### Fugl-Meyer Assessment of the Upper Extremity

**Figure 4:**
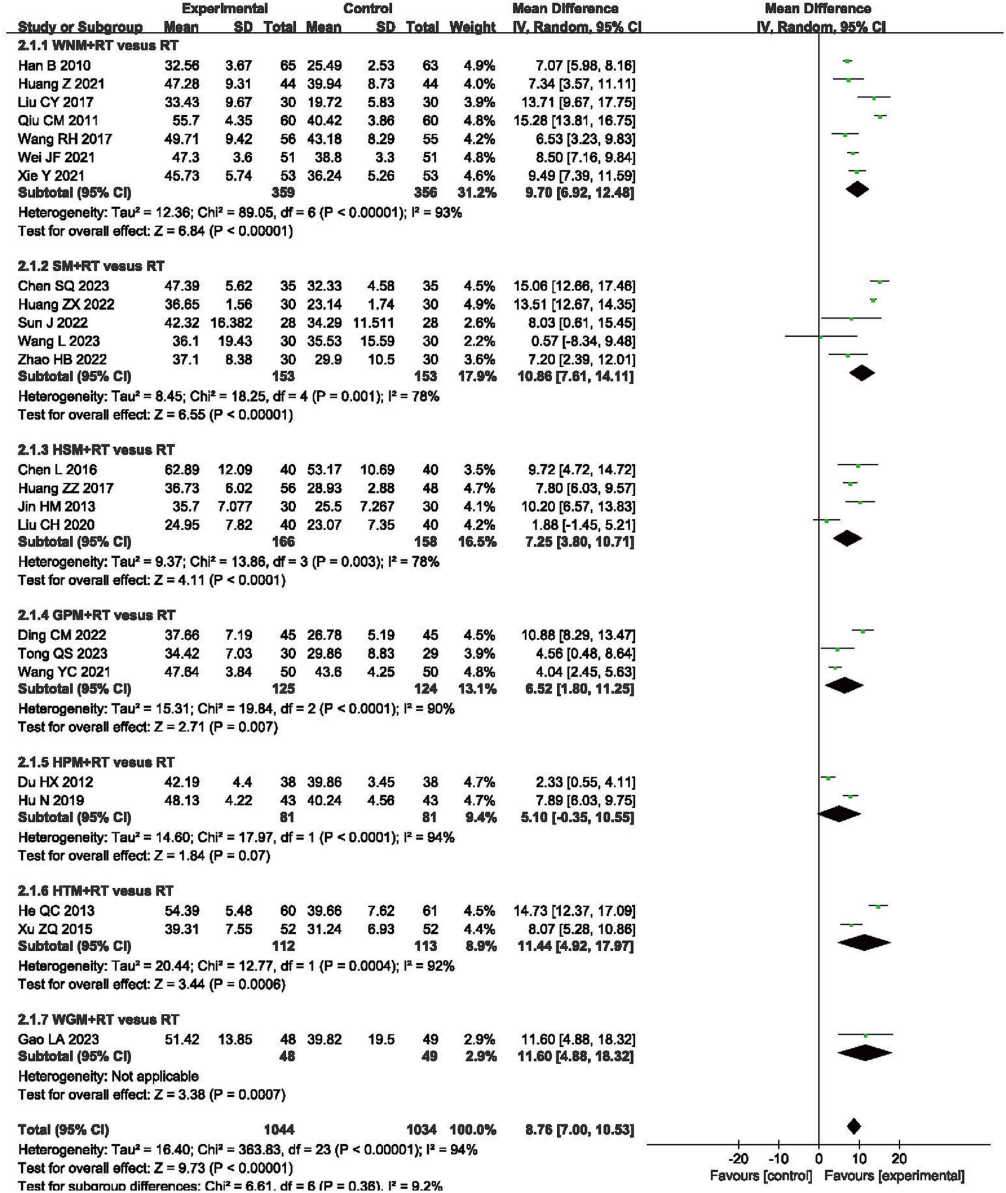
Forest plot of Fugl-Meyer Assessment of the Upper Extremity (FMA-UE) scale when comparing moxibustion plus RT (rehabilitation training) vs. RT alone.

Fugl-Meyer Assessment of the Upper Extremity (FMA-UE) scale is universally recognized for its utility in evaluating the upper extremity motor function of post-stroke patients, and was selected to assess differences in efficacy for moxibustion plus RT vs. RT alone in 24 trials that involved 2,078 patients (31–33, 35, 36, 39, 40, 43–45, 47, 49, 51–57, 59–63). The results showed that FMA-UE scores in the moxibustion plus RT groups exhibited greater improvement than those in the RT alone control groups (MD 8.76, 95% CI -7.00–10.53, *p* < 0.00001) with high heterogeneity (I^2^ = 94%, *p* < 0.00001, shown in Figure 4).

### Modified Barthel Index

**Figure 5:**
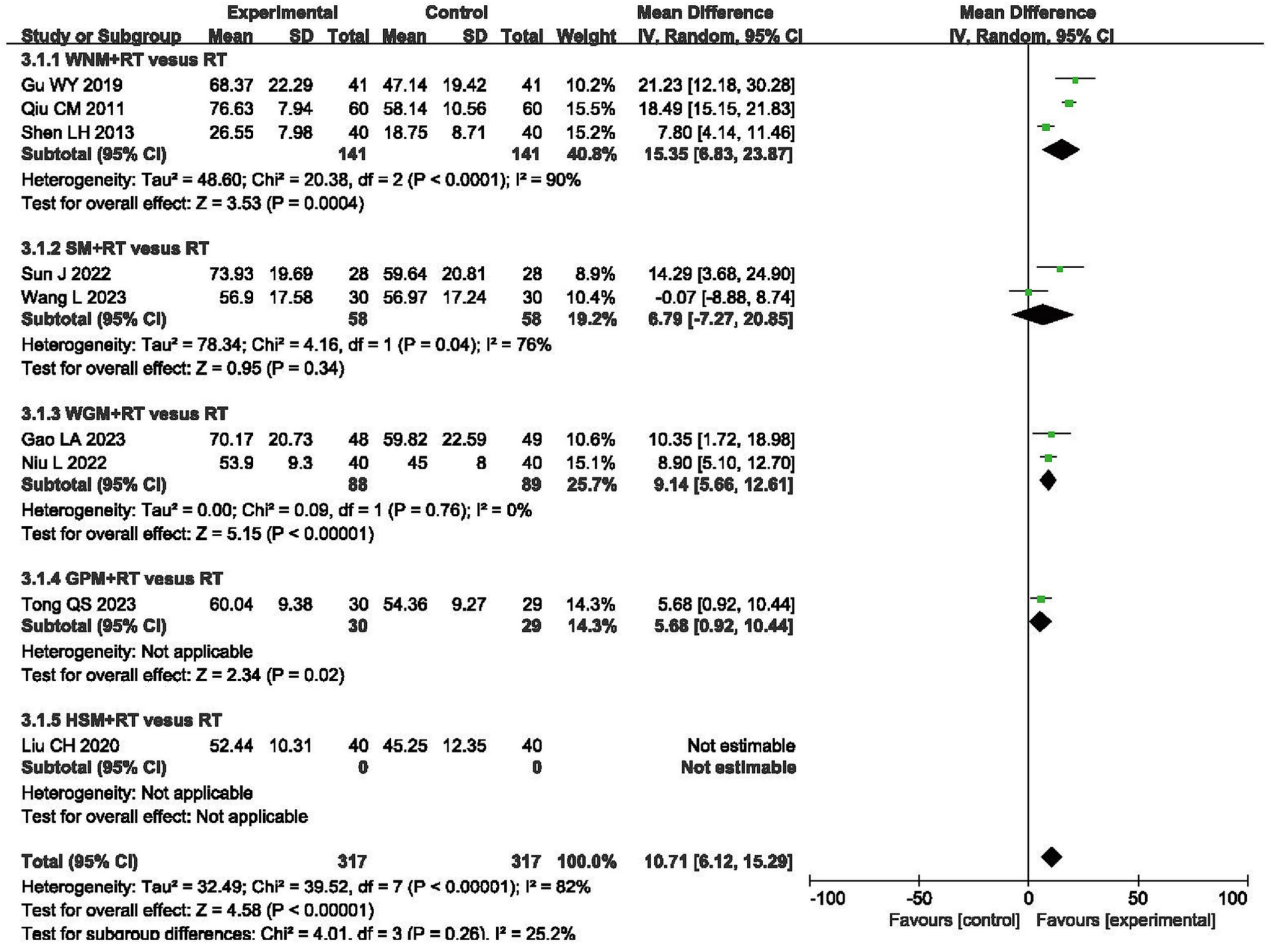
Forest plot of Modified Barthel Index (MBI) when comparing moxibustion plus RT (rehabilitation training) vs. RT alone.

Nine RCTs involving a total of 714 patients reported results from the Modified Barthel Index (MBI), one of the scales for evaluating the capacity for independently performing activities of daily living (ADLs) (32, 37, 46, 49, 58, 59, 61–63). The final results indicated that moxibustion plus RT achieved greater improvements on the MBI when compared to RT alone (MD 10.27, 95% CI 6.16–14.37, *p* < 0.00001) with high heterogeneity (I^2^ = 81%, *p* < 0.00001, shown in Figure 5).

### Barthel Index

**Figure 6:**
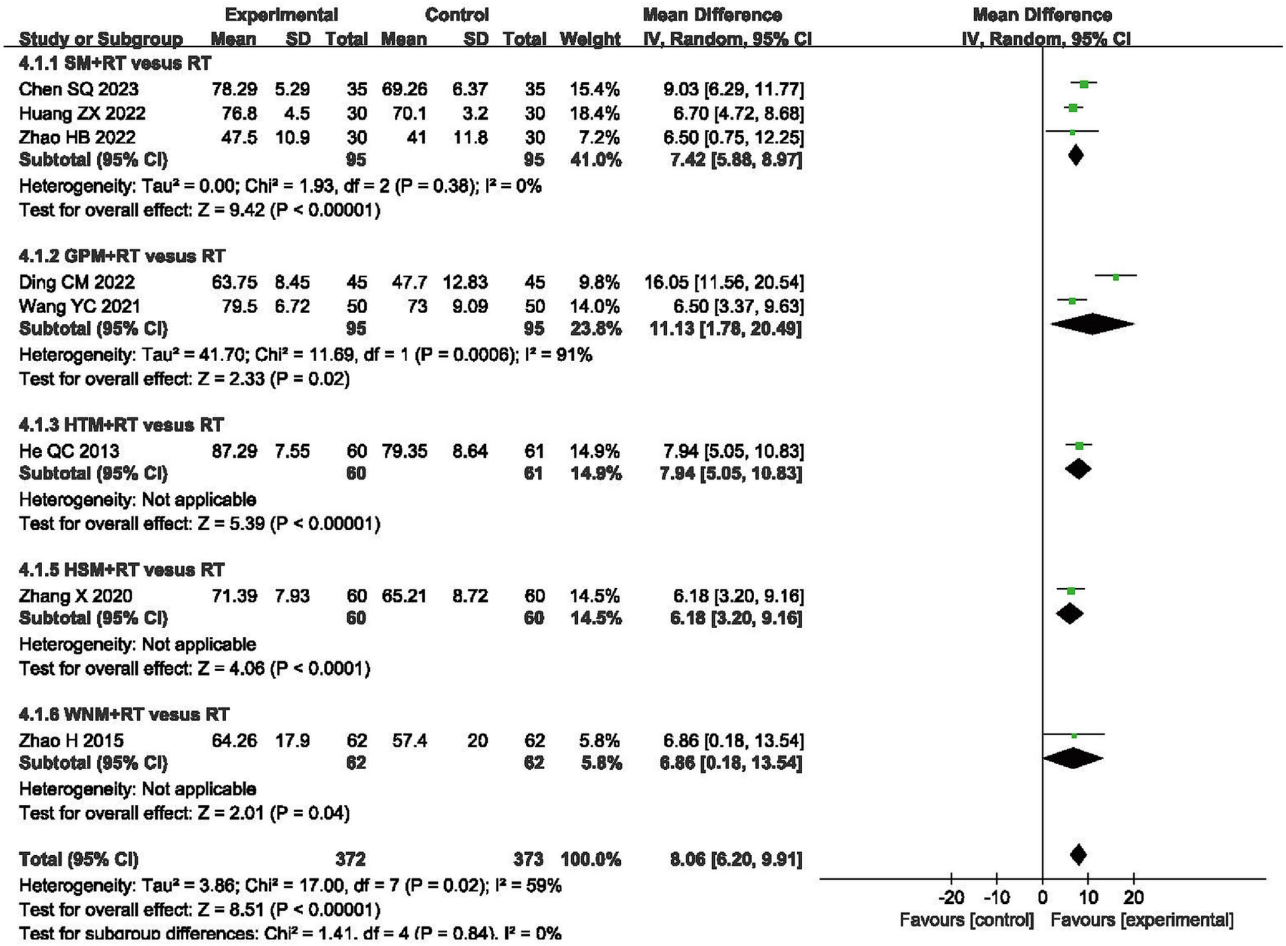
Forest plot of Barthel Index (BI) when comparing moxibustion plus RT (rehabilitation training) vs. RT alone.

A total of 745 patients from eight RCTs were evaluated using the Barthel index (BI) (35, 38, 50, 51, 55–57, 60). Meta-analysis revealed a greater increase in BI scores in the moxibustion plus RT groups, as compared to those observed in the RT alone groups (MD 8.06, 95% CI 6.20, 9.91, *p* < 0.00001) with high heterogeneity (I^2^ = 59%, *p* = 0.02, shown in Figure 6).

### National Institute of Health Stroke Scale

The National Institute of Health Stroke Scale (NIHSS) is also a measure of the ability to perform ADLs, with lower scores indicating a greater ability to perform these activities independently. Two trials, involving a total of 172 subjects, utilized this scale. The pooled MD obtained from applying a FE model showed a decrease in NIHSS scores in the moxibustion plus RT groups (MD -2.34, 95% CI -2.96– -1.72, *p* < 0.00001) with acceptable heterogeneity (I^2^ = 0%, *p* = 0.87, shown in Supplementary Figure 1).

### Total effective rates

The meta-analysis of total effective rates (TERs) totally included 1751 patients from 20 RCTs (31, 34–36, 40–44, 46–48, 50, 51, 53, 54, 58, 60–63). Even though TERs were evaluated by different scales (64–67), the ineffective rates were all evaluated by same standardizations in terms of no improvement on pain relieving or motor impairment or even worse conditions. Outcomes of meta-analysis indicated that the overall efficacy in experimental groups was better than control groups (RR 1.27, 95% CI 1.21–1.33, I^2^ = 0, *p* < 0.00001) with acceptable heterogeneity (I^2^ = 0%, *p* = 0.78, shown in Supplementary Figure 2).

### Adverse events

**Figure 7:**
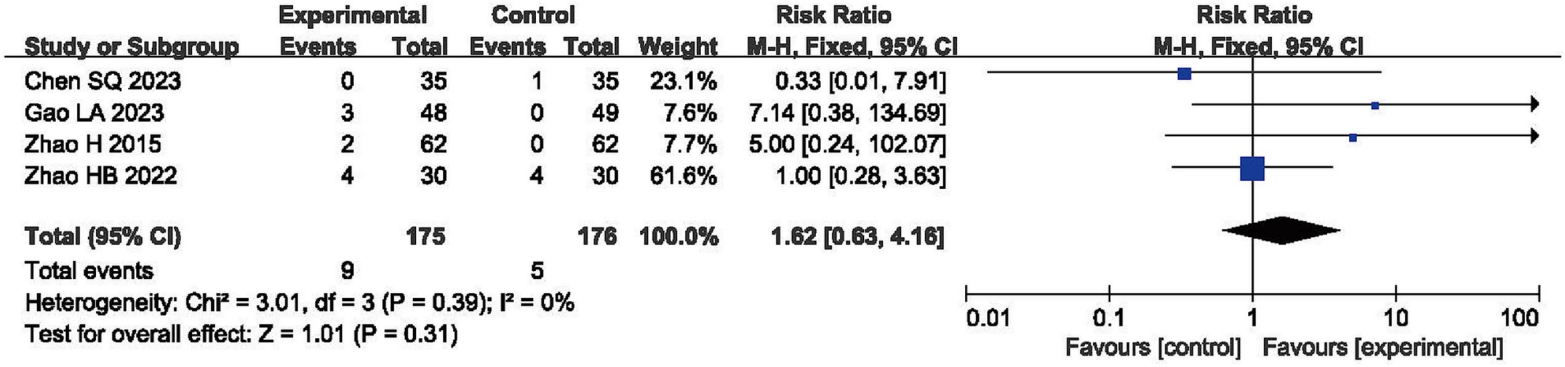
Forest plot of adverse events (AEs).

Four RCTs reported AEs, with no significant difference being observed in their incidence during comparisons between the experimental and control groups (RR = 1.62, 95% CI: 0.63, 4.16, *p* > 0.05) and with acceptable heterogeneity (I^2^ = 0%, *p* = 0.39, shown in Figure 7) (38, 55, 60, 61).

### Subgroup analysis

Because considerable heterogeneity was observed in the VAS, FMA-UE, MBI and BI scores, a subgroup analysis for each scale was conducted with consideration for the various types of moxibustion that were utilized, including SM, HSM, GPM, HPM, WNM, TFM, HTM and WGM. A sensitivity analysis was also conducted by excluding the most weighted study. Additionally, one single study was removed at a time, with analysis of the remaining trials being conducted in order to determine which study may have been responsible for disproportionately inducing heterogeneity and altering the final results.

The subgroup analysis of VAS scores revealed that intra-subgroup heterogeneity was acceptable among subgroups (I^2^ = 50, 0 and 0%), whereas inter-subgroup heterogeneity remained considerably high (I^2^ = 93.5%, shown in Figure 3).

Subgroup analysis using the FMA-UE showed that inter-subgroup heterogeneity was acceptable among subgroups (I^2^ = 9.2%), whereas intra-subgroup heterogeneity remained high (I^2^ = 93, 78, 78, 90%, 94 and 92%, shown in Figure 4).

Subgroup analysis using the MBI indicated that inter-subgroup heterogeneity was acceptable among subgroups (I^2^ = 5.9%), whereas some of intra-subgroup heterogeneity was still high (I^2^ = 90 and 76%, shown in Figure 5).

In terms of BI, the subgroup analysis demonstrated that inter-subgroup heterogeneity was acceptable among subgroups (I^2^ = 0%), whereas one instance of intra-subgroup heterogeneity remained high (I^2^ = 91%, shown in Figure 6).

### Sensitivity analysis

During subgroup analysis, little impact on the pooled MD was observed, as demonstrated by the results of the sensitivity analysis. However, we failed to identify the definitive cause of heterogeneity because the level of heterogeneity did not diminish upon removing any single study; details of this analysis are shown in Supplementary Table 5. Inconsistencies in moxibustion style, treatment durations, treatment frequencies and the length of treatment courses, as well as variations in acupoint selection, could all be potential causes for the heterogeneity that was observed.

### Publication bias and quality of evidence

**Figure 8:**
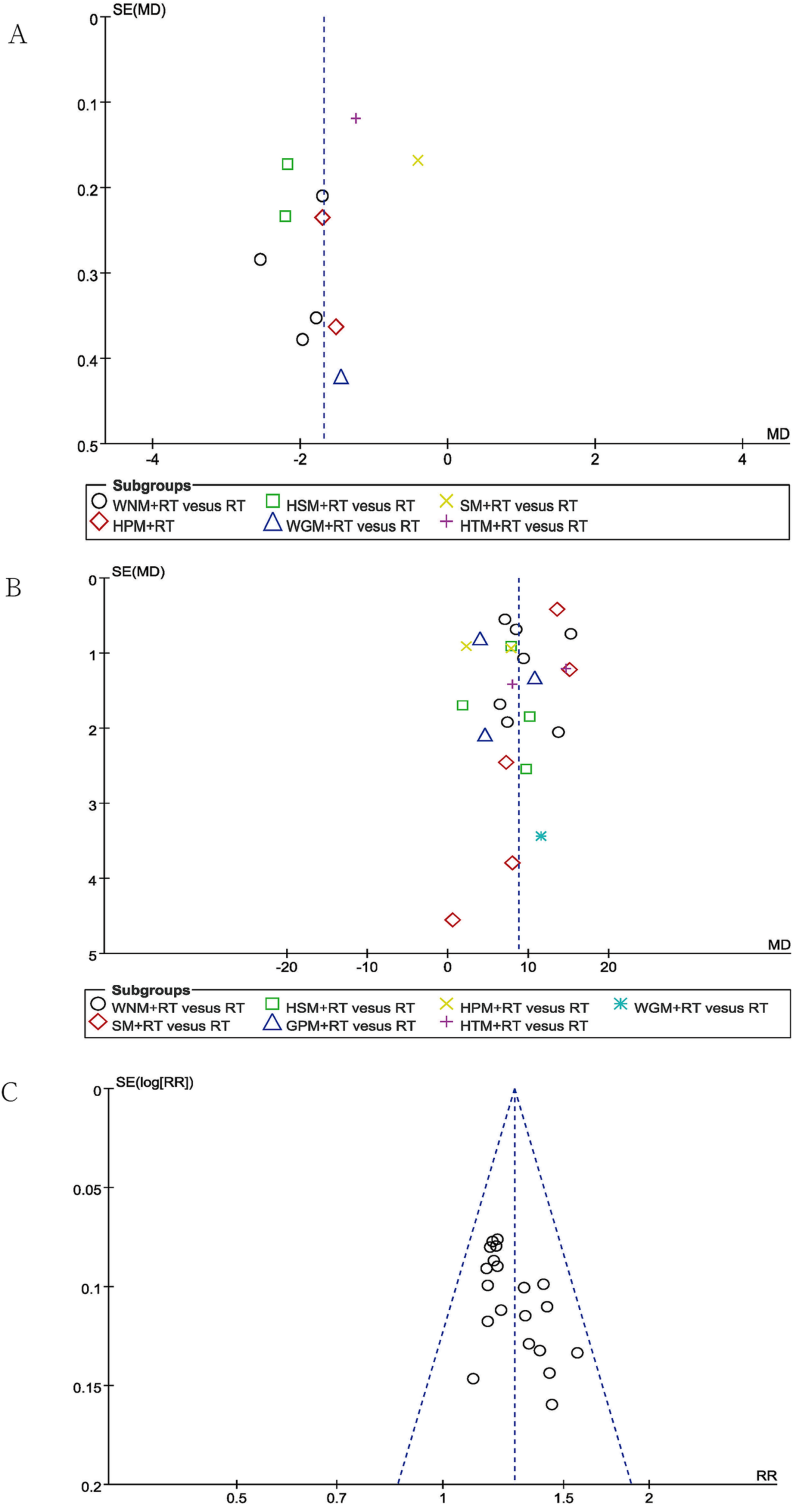
Funnel plots of VAS **(A)**, FMA-UE **(B)** and TERs **(C)**.

Publication bias was evaluated using funnel plots for VAS pain scores, the FMA-UE scores and TERs, which each included more than 10 studies. While the funnel plots for the TERs were roughly symmetrical, publication bias was clearly observed in the funnel plots examining the reports of changes in VAS pain scores and FMA-UE scores (shown in Figure 8). This finding could potentially be attributed to the absence of prospective clinical trial pre-registration practices, which can undermine the effort to demonstrate transparency in clinical research.

The overall strength of evidence gleaned from the included RCTs about the clinical efficacy of adjunctive moxibustion for treating post-stroke UE-PDMI was ultimately ranked as being of a “low” to “very low” level of certainty (shown in Table 3).

**Table 3:** Summary of findings and strength of evidence for outcomes.

Patient or population: patients with UE-PDMI Setting: hospitals in China				
Outcomes	Anticipated absolute effects^*^ (95% CI)		No. of participants (studies)	Certainty of the evidence (GRADE)
	Control experimental			
Moxibustion plus rehabilitation treatment compared to rehabilitation treatment for UE-PDMI *Intervention*: moxibustion plus rehabilitation treatment *Comparison*: rehabilitation treatment Pain disorder assessed by VAS (lower score less pain) Scale from: 0 to 10; follow up: 2–12 weeks	The mean VAS ranged from 1.07 to 6.13.	MD -1.86 lower (−2.08 lower to −1.28 lower)	1,055 (11 RCTs)	⨁⨁◯◯ LOW ^a,b^
Motor impairment assessed by FMA-UE (higher score better effect) Scale from: 0 to 66; follow up: 3–12 weeks	The mean FMA-UE ranged from 19.72 to 53.17.	MD 8.76 higher (7.00 higher to 10.53 higher)	2078 (24 RCTs)	⨁⨁◯◯ LOW ^a,b^
Motor impairment assessed by MBI (higher score better effect) Scale from: 0 to 100; follow up: 2–8 weeks	The mean MBI ranged from 18.75 to 59.64.	MD 10.27 higher (6.16 higher to 14.37 higher)	714 (9 RCTs)	⨁◯◯◯ VERY LOW ^a,b,d^
Motor impairment assessed by BI (higher score better effect) Scale from: 0 to 100; follow up: 2–12 weeks	The mean BI ranged from 47.7 to 79.35.	MD 8.06 higher (6.20 higher to 9.91 higher)	745 (8 RCTs)	⨁◯◯◯ VERY LOW ^a,b,d^
Performance of independent daily activities assessed by NIHSS (lower score better effect) Scale from: 0 to 42; follow up: 3–4 weeks	The mean NIHSS ranged from 10.82 to 13.50.	MD -2.34 lower (−2.96 lower to −1.72 lower)	172 (2 RCTs)	⨁◯◯◯ VERY LOW ^a,b,d^
Overall response rates assessed by TERs (higher score better effect) Scale from: 0 to 100%; follow up: 2–12 weeks	621/869 (71.5%)	RR 1.27 higher (1.21 higher to 1.33 higher)	1751 (20 RCTs)	⨁⨁◯◯ LOW ^a,d^

GRADE working group grades of evidence. *High certainty*: We are very confident that the true effect lies close to that of the estimate of the effect. *Moderate certainty*: We are moderately confident in the effect estimate: The true effect is likely to be close to the estimate of the effect, but there is a possibility that it is substantially different. *Low certainty*: Our confidence in the effect estimate is limited: The true effect may be substantially different from the estimate of the effect. *Very low certainty*: We have very little confidence in the effect estimate: The true effect is likely to be substantially different from the estimate of effect. ^*^The risk in the intervention group (and its 95% CI) is based on the assumed risk in the comparison group and the relative effect of the intervention (and its 95% CI). CI, Confidence interval; MD, Mean difference; RR, risk ratio; UE-PDMI, upper extremity pain disorder and motor impairment; VAS, visual analog scale; FMA-UE, Fugl-Meyer Assessment of the Upper Extremity; MBI, modified Barthel index; BI, Barthel index; NIHSS, National Institute of Health stroke scale; TERs, total effective rates.^a^Most of the RCTs were found to exhibit flaws in study protocol, or prospective registrations were unavailable.^b^Considerable heterogeneity (I^2^ > 50%) hindered meaningful data interpretation.^c^Total sample size in total was less than *n* = 400.^d^Blinding and allocation concealment was unclear among most of the RCTs, and an insufficient number of RCTs reported any evaluation for publication bias.

## Discussion

Overall, 32 RCTs involving a total of 2,814 patients were included for the final meta-analysis. The pooled data indicated that moxibustion serving as a monotherapy or as an adjuvant therapy could be beneficial for alleviating pain as measured by VAS scores and the independent performance of ADLs as measured by scores on the FMA-UE scale, MBI, BI, NIHSS and TERs for UE-PDMI recovery in patients with stage I post-stroke SHS. Furthermore, no significant differences in efficacy were observed during subgroup analysis. There was also no significant difference noted in the incidence of AEs when comparing experimental and control groups. Unfortunately, the certainty of evidence was low to very low because of poor methodological quality in trial design and substantial heterogeneity. However, this systematic review still provides an updated synthesis of the existing evidence from RCTs examining moxibustion for UE-PDMI.

Owing to its high rate of morbidity, UE-PDMI significantly jeopardizes post-stroke SHS survivors’ health (1). The potential pathophysiological mechanisms of these conditions have still yet to be fully elucidated, but may involve aberrant immune and inflammatory responses, degeneration within the central and peripheral nervous systems, vasomotor dysfunction, psychological factors and even genetically driven variations in the response to injury (10, 68). To date, the management of this condition mainly focuses on providing analgesia through pharmacological means, interventional pain management and rehabilitation, with the cumulative effect of these treatments unfortunately remaining unsatisfactory (10). Moxibustion, a cornerstone of TCM for millennia, has yet to be thoroughly studied regarding the breadth of the potential benefit that it may provide as a potential treatment for UE-PDMI (69). With moxibustion’s record of non-invasiveness, convenience of delivery, cost-effectiveness and efficacy for a broad range of related clinical indications, our research may provide novel insights into the potential clinical applications of moxibustion for this condition. A dearth of high-quality RCTs has necessitated the inclusion of RCTs involving low-quality methodology and reporting practices in this review. Nevertheless, even the less robust evidence gleaned from our review in support of moxibustion as an effective monotherapy or adjunctive therapy in treating UE-PDMI provides some propaedeutic insight that may guide and inspire future research.

Improvement was observed in the primary outcome measures of VAS pain scores and FMA-UE scores of motor function. Additionally, improvement was also observed in the secondary outcome measures of MBI, BI and NIHSS, which is especially noteworthy since a deterioration of upper extremity function tends to adversely affect the independent performance of ADLs, and consequently, any intervention that can be demonstrated to preserve this function would be invaluable for SHS patients suffering from UE-PDMI. All pooled results in our study have so far demonstrated multi-dimensional beneficial effects of moxibustion on UE-PDMI. However, as persistent pain and motor dysfunction progress into the chronic phase, psychological disorders such as elevated anxiety and depressive symptoms can often unavoidably occur (70). Our present review is unable to draw any conclusions regarding whether moxibustion may help alleviate such psychological disorders, because the currently available RCTs have yet to focus on evaluating this dimension of clinical care.

A detailed analysis of the included RCTs also reveals a great diversity of acupoints that were selected for the application of moxibustion. Half of the top 10 most frequently applied acupoints in these RCTs are located on Hand *Yangming* meridian, which is believed to play an essential role in activating the blood circulation and facilitating *Qi* circulation. This pattern of acupoint selection is consistent with TCM theory. Given that UE-PDMI is a chronically degenerative disease, most of the included RCTs favored a longer duration of intervention with more frequent treatment sessions and longer treatment courses to achieve and maintain desired clinical effects. With rising concerns about the risk of burn injury and the safety of smoke released during the process of administering moxibustion, we have paid special attention to the reporting of related AEs in these RCTs. We are pleased to note that the incidence of severe burns from burning moxa was reported to be rare, even with direct moxibustion. Additionally, the incidence of moxa smoke-related respiratory issues requiring medical aid was similarly reported to be very low. It is lamentable that the quality of moxa floss utilized, which directly correlates with the magnitude of positive clinical outcomes, was not mentioned in any of the included RCTs (71). Furthermore, because evaluating the differential effects of various forms of moxibustion delivery was not the focus of this review, RCTs involving direct comparisons of different styles of moxibustion were excluded. Some of these nuances of moxibustion delivery could potentially serve as fruitful avenues for research in future studies.

Because of obvious heterogeneities observed among the included studies, a RE model was noted to be frequently utilized. Heterogeneity remained high even with subgroup analysis taking the style of moxibustion as a stratified factor. As mentioned in the introduction part, there are seven different approaches of moxibustion included in this SR with direct and indirect patterns. Firstly, the seven distinct moxibustion techniques differ entirely in their operational procedures. Variations in moxa cone size specifications, differences in the quality of moxa wool, discrepancies in the distance from the skin during application, and diverse degrees of thermal stimulation to the local skin area collectively contribute to the high heterogeneity observed. Additional factors including the combination with acupuncture needles, various acupoints selected, and notably, certain techniques even incorporate herbal components as a conductive medium, further amplify procedural variations. These multidimensional differences in material composition, operational parameters, and adjunct therapeutic elements possibly constitute the principal sources of clinical heterogeneity in moxibustion interventions. Secondly, substantial variability exists in the demographic characteristics of enrolled patients across studies. Although all patients were diagnosed with either ischemic or hemorrhagic stroke, the mean age ranged from 46.27 to 68.40 years, representing an age span exceeding two decades. Younger patients generally exhibit superior neurorehabilitation outcomes compared to elder individuals due to age-related differences in neuroplasticity. Furthermore, marked discrepancies were observed in disease duration among studies, with mean post-stroke intervals varying from 18.2 days (earliest intervention) to 120 days (latest intervention). Earlier therapeutic intervention is clinically associated with more pronounced neurological improvement. Critically, moxibustion administration parameters demonstrated significant heterogeneity: single-session duration spanned 15 to 60 min; treatment frequency ranged from once weekly to daily applications; and total intervention cycles differed from 15 days (shortest) to 12 weeks (longest). The absence of authoritative expert guidelines or consensus has permitted this substantial divergence in core therapeutic variables, including temporal parameters, dosage intensity, and treatment scheduling, which ultimately constitutes a potential primary source of outcome heterogeneity in clinical evaluations of moxibustion efficacy for UE-PDMI.

## Strengths and limitations

This systematic review demonstrates notable methodological merits, foremost in advancing the current evidence base through contemporary evaluation of moxibustion’s safety-efficacy profile for UE-PDMI management, while simultaneously identifying critical knowledge gaps that necessitate prioritized investigation through hypothesis-driven clinical trials. A particular scholarly contribution lies in its development of a standardized multidimensional assessment framework incorporating validated biomarkers, functional metrics, and patient-reported outcomes – enabling systematic evaluation of moxibustion’s therapeutic benefits within neurorehabilitation contexts. The analytical approach was further strengthened through predefined subgroup analyses (stratified by intervention protocols and stroke subtypes) and methodological sensitivity analyses (assessing outcome stability across risk-of-bias tiers), which substantially strengthened the validity of key inferences. Nevertheless, several methodological constraints warrant acknowledgement. First and foremost, even with 32 RCTs eventually identified for inclusion, the methodological flaws and lack of transparency in study design were obvious, with a number of studies failing to provide important information or demonstrating ambiguities about the randomization, allocation, blinding and evaluation practices that were utilized. Only VAS pain scores and FMA-UE scores of motor function were utilized as the primary outcome measures in these RCTs included in this systematic review. Regretfully, the numerical rating scale (NRS), action research arm test (ARAT) and Wolf Motor Function Test were not applied, of which are more reliable and proper to measure the pain and motor dysfunction of upper limbs (72–74). All of these shortcomings introduce some potential bias, and the lack of high-quality RCTs unfortunately precludes our ability to draw definitive conclusions about the efficacy of moxibustion in this setting.

Secondly, we have been unable to formulate a definitive explanation for the considerable heterogeneity that has been observed to exist among the included RCTs. Subgroup analysis was initially conducted in an attempt to reduce heterogeneity by differentiating trials according to the various styles of moxibustion that they employed. Meanwhile, sensitivity analysis was also conducted in an attempt to label the potential cause(s) of the heterogeneity. Given that moxibustion is complex form of treatment, key factors may exist in the clinical nuances of how this modality is delivered, such as the quality of moxa floss, the size of moxa cones and sticks that are utilized, the combination of acupoints that are selected for treatment, and the frequency and duration of treatments, all of which varied considerably among the different RCTs. Additionally, with UE-PDMI in SHS being a comorbidity that commonly occurs after a stroke, the intricacy of its incompletely understood pathogenesis and pathophysiological mechanisms, its progression through various phases of chronicity, and the impact that the routine delivery of standard medical care may contribute to the healing process could also constitute factors that may be driving the significant heterogeneity that was observed in these studies.

Finally, our search was limited only to research articles that have been published in the English and Chinese languages, which could potentially have led to the exclusion of related literature that may have been published in other languages. All of the included RCTs were conducted in China, with the clinical rationale guiding the choice of moxibustion therapy being directly informed by TCM theory, and with the selection of acupoints being mainly based on regional proximity to the affected area and consideration of its overlapping meridian pathways. This geographic limitation may also restrict the consideration of other global perspectives on how this treatment could be effectively applied for the treatment of UE-PDMI in SHS, and may also in turn hinder the global recognition of this study’s contribution to the existing body of literature on this topic as well.

## Conclusion

The current evidence base remains insufficiently robust to endorse moxibustion as a standard therapeutic intervention for UE-PDMI in stage I post-stroke SHS. This systematic review reveals that diverse moxibustion modalities exhibit therapeutic potential in mitigating neuropathic pain components and facilitating sensorimotor recovery, while demonstrating favorable safety profiles. Notably, clinical outcomes appear independent of moxa delivery mechanisms, suggesting comparable efficacy across administration modalities. Collectively, these findings mandate methodologically rigorous RCTs featuring prospective registration, adequately powered sample sizes, and standardized outcome metrics to achieve three critical objectives: (1) Expand the evidence base through multi-center validation studies; (2) Generate definitive safety-efficacy data through biomarker-integrated trials; (3) Establish evidence-based clinical practice guidelines to optimize moxibustion protocol standardization (including dose–response parameters, treatment frequency, and duration). Such efforts would ultimately enable precision application of this TCM modality to optimize patient-centered care delivery in this neurologically vulnerable population.

## Data Availability

The original data presented in the study are included in the article/ Supplementary material, further inquiries can be directed to the corresponding authors.
