# Supplementary material for: Efficacy and safety of moxibustion treatment for upper extremity pain disorder and motor impairment in patients with stage I post-stroke shoulder-hand syndrome: a systematic review and meta-analysis of randomized controlled trials
Source: Front Neurol. 2025 May 23;16:1530069. doi: 10.3389/fneur.2025.1530069 (PMC12140999; doi:10.3389/fneur.2025.1530069)
Supplement: Supplementary file 1 [file Supplementary_file_1.docx]

**Supplementary Material**

**Search Strategy**

**1.Cochrane Central Register of Controlled Trials（CENTRAL, Clinical Trials Cochrane）ascribed to Cochrane Library**

Search manager with all limits set as “trials”

#1 stroke

#2 (brain stroke):ti,ab,kw

#3 (cerebral stroke):ti,ab,kw

#4 #1 OR #2 OR #3

#5 pain disorder

#6 pain

#7 (spasticity):ti,ab,kw

#8 (muscle cramp):ti,ab,kw

#9 (cramp):ti,ab,kw

#10 (motor impairment):ti,ab,kw

#11 (motor dysfunction):ti,ab,kw

#12 (shoulder hand syndrome):ti,ab,kw

#13 (complex regional pain syndrome):ti,ab,kw

#14 #5 OR #6 #7 OR #8 OR #9 OR #10 OR #11 OR #12 OR #13

#15 ("upper extremity"):ti,ab,kw

#16 (upper limb):ti,ab,kw

#17(shoulder):ti,ab,kw

#18 (hand):ti,ab,kw

#19 #15 OR #16 OR #17 OR 18

#20 moxibustion

#21 (moxa):ti,ab,kw

#22 (moxa floss):ti,ab,kw

#23 (moxa cone):ti,ab,kw

#24 #20 OR #21 OR #22 OR #23

#25 randomized controlled trial

#26 (clinical trial):ti,ab,kw

#27 (trial):ti,ab,kw

#28 (randomised controlled trial):ti,ab,kw

#29 #26 OR #27 OR #28

#30 #4 AND #14 AND #19 AND #24 AND #2

**2. WEB OF SCIENCE**

# Web of Science Search Strategy (v0.1)

# Searches:

1: ((TS=(stroke)) OR TS=(brain stroke)) OR TS=(cerebral stroke) and Preprint Citation Index (Exclude – Database)

2: ((TS=(upper extremity)) OR TS=(upper limb)) OR TS=(shoulder) OR TS=(hand) and Preprint Citation Index (Exclude – Database)

3: (((TS=(spasm)) OR TS=(spasticity)) OR TS=(muscle cramp)) OR TS=(pain) OR TS=(shoulder hand syndrome) OR TS=(motor impairment) and Preprint Citation Index (Exclude – Database)

4: (TS=(moxibustion)) OR TS=(moxa) OR TS=(moxa floss) OR TS=(moxa cone) and Preprint Citation Index (Exclude – Database)

5: ((TS=(clinical trial)) OR TS=(randomized controlled trial)) OR TS=(randomised controlled trial) and Preprint Citation Index (Exclude – Database)

6: #5 AND #4 AND #3 AND #2 AND #1 and Preprint Citation Index (Exclude – Database)

**3. Pubmed**

#1 "stroke"[MeSH Terms] OR "brain stroke"[Title/Abstract] OR "cerebral stroke"[Title/Abstract]

#2 "upper extremit*"[Title/Abstract] OR "upper limb"[Title/Abstract] OR "shoulder"[Title/Abstract] OR "hand"[Title/Abstract]

#3 "pain" [MeSH Terms] OR "pain disorder" [Title/Abstract] OR "spasticity"[Title/Abstract] OR "muscle cramp"[Title/Abstract] OR "cramp"[Title/Abstract] OR "motor impairment"[Title/Abstract] OR "motor dysfunction"[Title/Abstract] OR "shoulder hand syndrome"[Title/Abstract] OR "complex regional pain syndrome"[Title/Abstract]

#4 "moxibustion"[MeSH Terms] OR "moxa"[Title/Abstract] OR "moxa cone"[Title/Abstract] OR "moxa floss"[Title/Abstract]

#5 "randomized controlled trial"[MeSH Terms] OR "clinical trial"[Title/Abstract] OR "randomised controlled trial"[Title/Abstract] OR "trial"[Title/Abstract]

#1 AND #2 AND #3 AND #4 AND #5

**4. Embase**

#1 'stroke'/exp

#2 'pain'/exp

#3 'moxibustion'/exp

#4 'randomized controlled trial'/exp

#5 'brain stroke’: ti,ab,kw OR 'cerebral stroke':ti,ab,kw

#6 'upper extremit*’: ti,ab,kw OR 'upper limb':ti,ab,kw OR 'shoulder':ti,ab,kw OR 'hand':ti,ab,kw

#7 'pain disorder':ti,ab,kw OR 'spasticity':ti,ab,kw OR 'muscle cramp':ti,ab,kw OR 'cramp':ti,ab,kw OR 'motor impairment':ti,ab,kw OR 'motor dysfunction':ti,ab,kw OR 'shoulder hand syndrome':ti,ab,kw OR 'complex regional pain syndrome':ti,ab,kw

#8 'moxa’: ti,ab,kw OR 'moxa cone':ti,ab,kw OR 'moxa floss':ti,ab,kw

#9 'clinical trial’: ti,ab,kw OR 'randomised controlled trial':ti,ab,kw OR 'trial':ti,ab,kw

#10 #1 OR #5 OR #6

#11 #2 OR #7

#12 #3 OR #8

#13 #4 OR #9

#14 #10 AND #11 AND #12 AND #13

**5. CNKI**

(TKA = '脑卒中' OR TKA = '中风' OR TKA = '卒中' OR TKA = '脑梗死' OR TKA = '脑出血' )AND (TKA = '痉挛' OR TKA = '偏瘫' OR TKA = '疼痛' OR TKA = '运动障碍' OR TKA = '综合征') AND (TKA = '上肢' OR TKA = '手' OR TKA = '肩' )AND (TKA = '艾灸' OR TKA = '灸') AND (TKA = '临床' OR TKA = '试验' OR TKA = '随机'OR TKA = '随机对照试验')

**6.VIP**

（M=脑卒中 OR M=中风 OR M=卒中 OR M=脑梗死 OR M=脑出血) AND (M=疼痛 OR M=痉挛 OR M=偏瘫 OR M=硬瘫 OR M=运动障碍) AND (M=上肢 OR M=肩 OR M=手 OR M=综合征) AND (M=艾灸 OR M=灸) AND (M=临床 OR M=临床试验OR M=随机OR M=随机对照试验)

**7.Wanfang**

((“脑卒中”or“中风”or “卒中”) and (“疼痛”or “偏瘫”or “痉挛”or “运动障碍”) and (“上肢”or “肩 ”or “手“) and ("艾灸" or "灸") and ("临床" or "临床试验" or "随机" or "随机对照试验"))

**8.SinoMed**

1) "脑卒中"[常用字段:智能] OR "中风"[常用字段:智能] AND "卒中"[常用字段:智能]

2) "疼痛"[常用字段:智能] OR "痉挛"[常用字段:智能] OR "偏瘫"[常用字段:智能] OR "运动障碍"[常用字段:智能]

3) "上肢"[常用字段:智能] OR "肩"[常用字段:智能] OR "手"[常用字段:智能]

4) "艾灸"[常用字段:智能] OR "灸"[常用字段:智能]

5) "临床"[常用字段:智能] OR "临床试验"[常用字段:智能] OR "随机"[常用字段:智能] OR "随机对照"[常用字段:智能] OR "随机对照试验"[常用字段:智能]

6) (#5) AND (#4) AND (#3) AND (#2) AND (#1) **Supplementary Material**

**Table 1 Detailed information about different types of moxibustion**

| **Intervention** | **Type** | **Explanation** |
| --- | --- | --- |
| Suspended moxibustion (SM) | Indirect moxibustion | Ignited moxa floss was suspended over acupoints at a certain distance with heat radiating to the skin, causing a warm sensation on the skin surface. |
| Ginger-partitioned moxibustion (GPM) | Indirect moxibustion | Ignited moxa floss was placed on top of sliced fresh ginger, 3-4 millimeter in thickness with small holes to facilitate the heat conduction. |
| Herbal-partitioned moxibustion (HPM) | Indirect moxibustion | Applying herbal powder as insulating materials, ignited moxa floss was placed on top. |
| Heat-sensitive moxibustion (HPM) | Indirect moxibustion | Ignited moxa floss were suspended only on sensitive acupoints, which could generate the penetration and radiation of the heat sensations. |
| Warm needling moxibustion (WNM) | Indirect moxibustion | Ignited moxa floss with some distance from the skin were wrapped on the needle handle during the needle retention process, conducting the heat downwards through the body of needles. |
| Thunder-fire moxibustion (TFM) | Direct moxibustion | Ignited moxa floss combined with some herbal powder, which was wrapped up with several layers of cloth, was pressed directly on acupoints. |
| Herbal thread moxibustion (HTM) | Direct moxibustion | Cotton thread was immersed into some certain herbal powder mixed with Chinese liquor. Twist 4-6 strands of cotton thread tightly into a rope with a thickness of about 1.5-2.0mm after complete dryness. After rubbing the cotton thread, smooth it with yellow wax for later use. Ignited herbal thread was pressed directly on regional skin. |
| Wheat-grain moxibustion (WGM) | Direct moxibustion | Moxa floss was kneaded into a size similar to wheat grains, and directly placed on acupoints after ignition. And replace the burnt-out ashes with newly ignited moxa floss for certain times. |

**Supplementary Material**

**Table 2. Overall quality Rating by the CONSORT guideline (n = 32)**

| **Criteria** | **Description** | **Number of positive trials (n)** | **Percentage**  **(%)** |
| --- | --- | --- | --- |
| **Title and abstract** | 1a Identification as a randomised trial in the title | 2 | 6.25 |
|  | 1b Structured summary of trial design, methods, results, and conclusions | 27 | 83.38 |
| **Introduction** | | | |
| Background and objectives | 2a Scientific background and explanation of rationale | 25 | 78.12 |
|  | 2b Specific objectives or hypotheses | 13 | 40.63 |
| **Methods** | | | |
| Trial design | 3a Description of trial design (such as parallel, factorial) including allocation ratio | 5 | 15.63 |
|  | 3b Important changes to methods after trial commencement (such as eligibility criteria), with reasons | 0 | 0 |
| Participants | 4a Eligibility criteria for participants | 30 | 93.75 |
|  | 4b Settings and locations where the data were collected | 27 | 84.38 |
| Interventions | 5 The interventions for each group with sufficient details to allow replication, including how and when they were actually administered | 32 | 100 |
| Outcomes | 6a Completely defined prespecified primary and secondary outcome measures, including how and when they were assessed | 12 | 37.50 |
|  | 6b Any changes to trial outcomes after the trial commenced, with reasons | 0 | 0 |
| Sample size | 7a How sample size was determined | 0 | 0 |
|  | 7b When applicable, explanation of any interim analyses and stopping guidelines | 0 | 0 |
| Randomisation | 8a Method used to generate the random allocation sequence | 24 | 75 |
|  | 8b Type of randomisation; details of any restriction (such as blocking and block size) | 5 | 15.63 |
| Allocation concealment | 9 Mechanism used to implement the random allocation sequence (such as sequentially numbered containers), describing any steps taken to conceal the sequence until interventions were assigned | 4 | 12.50 |
| Implementation | 10 Who generated the random allocation sequence, who enrolled participants, and who assigned participants to interventions | 4 | 12.50 |
| Blinding | 11a If done, who was blinded after assignment to interventions (for example, participants, care providers, those assessing outcomes) and how | 1 | 3.13 |
|  | 11b If relevant, description of the similarity of interventions | 1 | 3.13 |
| Statistical methods | 12a Statistical methods used to compare groups for primary and secondary outcomes | 30 | 93.75 |
|  | 12b Methods for additional analyses, such as subgroup analyses and adjusted analyses | 0 | 0 |
| **Results** | | | |
| Flow chart | 13a For each group, the numbers of participants who were randomly assigned, received intended treatment, and were analysed for the primary outcome | 32 | 100 |
|  | 13b For each group, losses and exclusions after randomisation, together with reasons | 12 | 37.50 |
| Recruitment | 14a Dates defining the periods of recruitment and follow-up | 30 | 93.75 |
|  | 14b Why the trial ended or was stopped | 0 | 0 |
| Baseline data | 15 A table showing baseline demographic and clinical characteristics for each group | 15 | 46.88 |
| Intent-to-treat analysis | 16 For each group, number of participants (denominator) included in each analysis and whether the analysis was by original assigned groups | 0 | 0 |
| Outcomes and estimation | 17a For each primary and secondary outcome, results for each group, and the estimated effect size and its precision (such as 95% confidence interval) | 32 | 100 |
|  | 17b For binary outcomes, presentation of both absolute and relative effect sizes is recommended | 0 | 0 |
| Ancillary analyses | 18 Results of any other analyses performed, including subgroup analyses and adjusted analyses, distinguishing pre-specified from exploratory | 0 | 0 |
| Harms | 19 All important harms or unintended effects in each group (for specific guidance see CONSORT for harms) | 4 | 12.5 |
| **Discussion** | | | |
| Limitations | 20 Trial limitations, addressing sources of potential bias, imprecision, and, if relevant, multiplicity of analyses | 7 | 21.88 |
| Generalisability | 21 Generalisability (external validity, applicability) of the trial findings | 6 | 18.75 |
| Interpretation | 22 Interpretation consistent with results, balancing benefits and harms, and considering other relevant evidence | 29 | 90.63 |
| **Other information** | | | |
| Registration | 23 Registration number and name of trial registry | 0 | 0 |
| Protocol | 24 Where the full trial protocol can be accessed, if available | 0 | 0 |
| Funding | 25 Sources of funding and other support (such as supply of drugs), role of funders | 11 | 34.38 |

**Supplementary Material Table 3**. **The assessment of risk of bias**

**Chang WY 2013**

| **Bias** | **Authors' judgement** | **Support for judgement** |
| --- | --- | --- |
| Random sequence generation (selection bias) | Unclear risk | The specific method of randomization which was chosen was not reported. |
| Allocation concealment (selection bias) | Unclear risk | Not mentioned. |
| Blinding of participants and personnel (performance bias) | Unclear risk | Not mentioned. |
| Blinding of outcome assessment (detection bias) | Unclear risk | Not mentioned. |
| Incomplete outcome data (attrition bias) | Low risk | No missing data. |
| Selective reporting (reporting bias) | High risk | Only total effective rate was reported. |
| Other bias | Low risk | No other sources of bias. |

**Chen L 2016**

| **Bias** | **Authors' judgement** | **Support for judgement** |
| --- | --- | --- |
| Random sequence generation (selection bias) | Low risk | Software was applied to generate the randomized number. |
| Allocation concealment (selection bias) | Unclear risk | Not mentioned. |
| Blinding of participants and personnel (performance bias) | Unclear risk | Not mentioned. |
| Blinding of outcome assessment (detection bias) | Unclear risk | Not mentioned. |
| Incomplete outcome data (attrition bias) | Low risk | No missing data. |
| Selective reporting (reporting bias) | Low risk | All outcomes had been reported. |
| Other bias | Low risk | No other sources of bias. |

**Chen SQ 2023**

| **Bias** | **Authors' judgement** | **Support for judgement** |
| --- | --- | --- |
| Random sequence generation (selection bias) | Low risk | A random number table was applied. |
| Allocation concealment (selection bias) | Unclear risk | Not mentioned. |
| Blinding of participants and personnel (performance bias) | Unclear risk | Not mentioned. |
| Blinding of outcome assessment (detection bias) | Unclear risk | Not mentioned. |
| Incomplete outcome data (attrition bias) | Low risk | No missing data. |
| Selective reporting (reporting bias) | Low risk | All outcomes had been reported. |
| Other bias | Low risk | No other sources of bias. |

**Chen XB 2017**

| **Bias** | **Authors' judgement** | **Support for judgement** |
| --- | --- | --- |
| Random sequence generation (selection bias) | Low risk | A random number table was applied. |
| Allocation concealment (selection bias) | Unclear risk | Not mentioned. |
| Blinding of participants and personnel (performance bias) | Unclear risk | Not mentioned. |
| Blinding of outcome assessment (detection bias) | Unclear risk | Not mentioned. |
| Incomplete outcome data (attrition bias) | Low risk | No missing data. |
| Selective reporting (reporting bias) | Low risk | All outcomes had been reported. |
| Other bias | Low risk | No other sources of bias. |

**Ding CM 2022**

| **Bias** | **Authors' judgement** | **Support for judgement** |
| --- | --- | --- |
| Random sequence generation (selection bias) | Low risk | A random number table was applied. |
| Allocation concealment (selection bias) | Unclear risk | Not mentioned. |
| Blinding of participants and personnel (performance bias) | Unclear risk | Not mentioned. |
| Blinding of outcome assessment (detection bias) | Unclear risk | Not mentioned. |
| Incomplete outcome data (attrition bias) | Low risk | No missing data. |
| Selective reporting (reporting bias) | Low risk | All outcomes had been reported. |
| Other bias | Low risk | No other sources of bias. |

**Du HX 2012**

| **Bias** | **Authors' judgement** | **Support for judgement** |
| --- | --- | --- |
| Random sequence generation (selection bias) | Low risk | A random number table was applied. |
| Allocation concealment (selection bias) | Unclear risk | Not mentioned. |
| Blinding of participants and personnel (performance bias) | Unclear risk | Not mentioned. |
| Blinding of outcome assessment (detection bias) | Unclear risk | Not mentioned. |
| Incomplete outcome data (attrition bias) | Low risk | No missing data. |
| Selective reporting (reporting bias) | Low risk | All outcomes had been reported. |
| Other bias | Low risk | No other sources of bias. |

**Gao LA 2023**

| **Bias** | **Authors' judgement** | **Support for judgement** |
| --- | --- | --- |
| Random sequence generation (selection bias) | Low risk | A random number table was applied. |
| Allocation concealment (selection bias) | Unclear risk | Not mentioned. |
| Blinding of participants and personnel (performance bias) | Unclear risk | Not mentioned. |
| Blinding of outcome assessment (detection bias) | Unclear risk | Not mentioned. |
| Incomplete outcome data (attrition bias) | High risk | 3 dropouts without a proper intention-to-treat analysis. |
| Selective reporting (reporting bias) | Low risk | All outcomes had been reported. |
| Other bias | Low risk | No other sources of bias. |

**Gu WY 2019**

| **Bias** | **Authors' judgement** | **Support for judgement** |
| --- | --- | --- |
| Random sequence generation (selection bias) | Low risk | A random number table was applied. |
| Allocation concealment (selection bias) | Unclear risk | Not mentioned. |
| Blinding of participants and personnel (performance bias) | Unclear risk | Not mentioned. |
| Blinding of outcome assessment (detection bias) | Unclear risk | Not mentioned. |
| Incomplete outcome data (attrition bias) | Low risk | No missing data. |
| Selective reporting (reporting bias) | Low risk | All outcomes had been reported. |
| Other bias | Low risk | No other sources of bias. |

**Han B 2010**

| **Bias** | **Authors' judgement** | **Support for judgement** |
| --- | --- | --- |
| Random sequence generation (selection bias) | Low risk | A random number table was applied. |
| Allocation concealment (selection bias) | Unclear risk | Not mentioned. |
| Blinding of participants and personnel (performance bias) | Unclear risk | Not mentioned. |
| Blinding of outcome assessment (detection bias) | Unclear risk | Not mentioned. |
| Incomplete outcome data (attrition bias) | Low risk | No missing data. |
| Selective reporting (reporting bias) | Low risk | All outcomes had been reported. |
| Other bias | Low risk | No other sources of bias. |

**He QC 2013**

| **Bias** | **Authors' judgement** | **Support for judgement** |
| --- | --- | --- |
| Random sequence generation (selection bias) | Unclear risk | The specific method of randomization which was chosen was not reported. |
| Allocation concealment (selection bias) | Unclear risk | Not mentioned. |
| Blinding of participants and personnel (performance bias) | Unclear risk | Not mentioned. |
| Blinding of outcome assessment (detection bias) | Unclear risk | Not mentioned. |
| Incomplete outcome data (attrition bias) | High risk | 2 dropouts without a proper intention-to-treat analysis. |
| Selective reporting (reporting bias) | Low risk | All outcomes had been reported. |
| Other bias | Low risk | No other sources of bias. |

**Huang Z 2021**

| **Bias** | **Authors' judgement** | **Support for judgement** |
| --- | --- | --- |
| Random sequence generation (selection bias) | Low risk | A random number table was applied. |
| Allocation concealment (selection bias) | Unclear risk | Not mentioned. |
| Blinding of participants and personnel (performance bias) | Unclear risk | Not mentioned. |
| Blinding of outcome assessment (detection bias) | Unclear risk | Not mentioned. |
| Incomplete outcome data (attrition bias) | Low risk | No missing data. |
| Selective reporting (reporting bias) | Low risk | All outcomes had been reported. |
| Other bias | Low risk | No other sources of bias. |

**Huang ZX 2022**

| **Bias** | **Authors' judgement** | **Support for judgement** |
| --- | --- | --- |
| Random sequence generation (selection bias) | High risk | No randomization method was applied. |
| Allocation concealment (selection bias) | Unclear risk | Not mentioned. |
| Blinding of participants and personnel (performance bias) | Unclear risk | Not mentioned. |
| Blinding of outcome assessment (detection bias) | Unclear risk | Not mentioned. |
| Incomplete outcome data (attrition bias) | Low risk | No missing data. |
| Selective reporting (reporting bias) | Low risk | All outcomes had been reported. |
| Other bias | Low risk | No other sources of bias. |

**Huang ZZ 2017**

| **Bias** | **Authors' judgement** | **Support for judgement** |
| --- | --- | --- |
| Random sequence generation (selection bias) | High risk | No randomization method was applied. |
| Allocation concealment (selection bias) | Unclear risk | Not mentioned. |
| Blinding of participants and personnel (performance bias) | Unclear risk | Not mentioned. |
| Blinding of outcome assessment (detection bias) | Unclear risk | Not mentioned. |
| Incomplete outcome data (attrition bias) | Low risk | No missing data. |
| Selective reporting (reporting bias) | Low risk | All outcomes had been reported. |
| Other bias | Low risk | No other sources of bias. |

**Hu N 2019**

| **Bias** | **Authors' judgement** | **Support for judgement** |
| --- | --- | --- |
| Random sequence generation (selection bias) | Low risk | A random number table was applied. |
| Allocation concealment (selection bias) | Unclear risk | Not mentioned. |
| Blinding of participants and personnel (performance bias) | Unclear risk | Not mentioned. |
| Blinding of outcome assessment (detection bias) | Unclear risk | Not mentioned. |
| Incomplete outcome data (attrition bias) | Low risk | No missing data. |
| Selective reporting (reporting bias) | Low risk | All outcomes had been reported. |
| Other bias | Low risk | No other sources of bias. |

**Jin HM 2013**

| **Bias** | **Authors' judgement** | **Support for judgement** |
| --- | --- | --- |
| Random sequence generation (selection bias) | Low risk | A random number table was applied. |
| Allocation concealment (selection bias) | Unclear risk | Not mentioned. |
| Blinding of participants and personnel (performance bias) | Unclear risk | Not mentioned. |
| Blinding of outcome assessment (detection bias) | Unclear risk | Not mentioned. |
| Incomplete outcome data (attrition bias) | Low risk | No missing data. |
| Selective reporting (reporting bias) | Low risk | All outcomes had been reported. |
| Other bias | Low risk | No other sources of bias. |

**Liu CH 2020**

| **Bias** | **Authors' judgement** | **Support for judgement** |
| --- | --- | --- |
| Random sequence generation (selection bias) | High risk | No randomization method was applied. |
| Allocation concealment (selection bias) | Unclear risk | Not mentioned. |
| Blinding of participants and personnel (performance bias) | Unclear risk | Not mentioned. |
| Blinding of outcome assessment (detection bias) | Unclear risk | Not mentioned. |
| Incomplete outcome data (attrition bias) | Low risk | No missing data. |
| Selective reporting (reporting bias) | Low risk | All outcomes had been reported. |
| Other bias | Low risk | No other sources of bias. |

**Liu CY 2017**

| **Bias** | **Authors' judgement** | **Support for judgement** |
| --- | --- | --- |
| Random sequence generation (selection bias) | Low risk | A random number table was applied. |
| Allocation concealment (selection bias) | Unclear risk | Not mentioned. |
| Blinding of participants and personnel (performance bias) | Unclear risk | Not mentioned. |
| Blinding of outcome assessment (detection bias) | Unclear risk | Not mentioned. |
| Incomplete outcome data (attrition bias) | Low risk | No missing data. |
| Selective reporting (reporting bias) | Low risk | All outcomes had been reported. |
| Other bias | Low risk | No other sources of bias. |

**Li YQ 2020**

| **Bias** | **Authors' judgement** | **Support for judgement** |
| --- | --- | --- |
| Random sequence generation (selection bias) | High risk | No randomization method was applied. |
| Allocation concealment (selection bias) | Unclear risk | Not mentioned. |
| Blinding of participants and personnel (performance bias) | Unclear risk | Not mentioned. |
| Blinding of outcome assessment (detection bias) | Unclear risk | Not mentioned. |
| Incomplete outcome data (attrition bias) | Low risk | No missing data. |
| Selective reporting (reporting bias) | Low risk | All outcomes had been reported. |
| Other bias | Low risk | No other sources of bias. |

**Niu L 2022**

| **Bias** | **Authors' judgement** | **Support for judgement** |
| --- | --- | --- |
| Random sequence generation (selection bias) | Low risk | SPSS Software was applied to generate the randomized number. |
| Allocation concealment (selection bias) | Low risk | Random numbers and group assigned were kept in sealed opaque envelopes. |
| Blinding of participants and personnel (performance bias) | Unclear risk | Not mentioned. |
| Blinding of outcome assessment (detection bias) | Unclear risk | Not mentioned. |
| Incomplete outcome data (attrition bias) | Low risk | No missing data. |
| Selective reporting (reporting bias) | Low risk | All outcomes had been reported. |
| Other bias | Low risk | No other sources of bias. |

**Qiu CM 2011**

| **Bias** | **Authors' judgement** | **Support for judgement** |
| --- | --- | --- |
| Random sequence generation (selection bias) | Low risk | A random number table was applied. |
| Allocation concealment (selection bias) | Unclear risk | Not mentioned. |
| Blinding of participants and personnel (performance bias) | Unclear risk | Not mentioned. |
| Blinding of outcome assessment (detection bias) | Unclear risk | Not mentioned. |
| Incomplete outcome data (attrition bias) | Low risk | No missing data. |
| Selective reporting (reporting bias) | Low risk | All outcomes had been reported. |
| Other bias | Low risk | No other sources of bias. |

**Shen LH 2013**

| **Bias** | **Authors' judgement** | **Support for judgement** |
| --- | --- | --- |
| Random sequence generation (selection bias) | Unclear risk | The specific method of randomization which was chosen was not reported. |
| Allocation concealment (selection bias) | Unclear risk | Not mentioned. |
| Blinding of participants and personnel (performance bias) | Unclear risk | Not mentioned. |
| Blinding of outcome assessment (detection bias) | Unclear risk | Not mentioned. |
| Incomplete outcome data (attrition bias) | Low risk | No missing data. |
| Selective reporting (reporting bias) | Low risk | All outcomes had been reported. |
| Other bias | Low risk | No other sources of bias. |

**Sun J 2022**

| **Bias** | **Authors' judgement** | **Support for judgement** |
| --- | --- | --- |
| Random sequence generation (selection bias) | Low risk | A random number table was applied. |
| Allocation concealment (selection bias) | Unclear risk | Not mentioned. |
| Blinding of participants and personnel (performance bias) | Unclear risk | Not mentioned. |
| Blinding of outcome assessment (detection bias) | Unclear risk | Not mentioned. |
| Incomplete outcome data (attrition bias) | Low risk | No missing data. |
| Selective reporting (reporting bias) | Low risk | All outcomes had been reported. |
| Other bias | Low risk | No other sources of bias. |

**Tong QS 2023**

| **Bias** | **Authors' judgement** | **Support for judgement** |
| --- | --- | --- |
| Random sequence generation (selection bias) | Low risk | A random number table was applied. |
| Allocation concealment (selection bias) | Unclear risk | Not mentioned. |
| Blinding of participants and personnel (performance bias) | Unclear risk | Not mentioned. |
| Blinding of outcome assessment (detection bias) | Low risk | Outcome assessment staff was blinded to the allocation. |
| Incomplete outcome data (attrition bias) | Low risk | No missing data. |
| Selective reporting (reporting bias) | Low risk | All outcomes had been reported. |
| Other bias | Low risk | No other sources of bias. |

**Wang L 2023**

| **Bias** | **Authors' judgement** | **Support for judgement** |
| --- | --- | --- |
| Random sequence generation (selection bias) | Low risk | A random number table was applied. |
| Allocation concealment (selection bias) | Unclear risk | Not mentioned. |
| Blinding of participants and personnel (performance bias) | Unclear risk | Not mentioned. |
| Blinding of outcome assessment (detection bias) | Unclear risk | Not mentioned. |
| Incomplete outcome data (attrition bias) | Low risk | No missing data. |
| Selective reporting (reporting bias) | Low risk | All outcomes had been reported. |
| Other bias | Low risk | No other sources of bias. |

**Wang RH 2017**

| **Bias** | **Authors' judgement** | **Support for judgement** |
| --- | --- | --- |
| Random sequence generation (selection bias) | Low risk | A random number table was applied. |
| Allocation concealment (selection bias) | Unclear risk | Not mentioned. |
| Blinding of participants and personnel (performance bias) | Unclear risk | Not mentioned. |
| Blinding of outcome assessment (detection bias) | Unclear risk | Not mentioned. |
| Incomplete outcome data (attrition bias) | Low risk | No missing data. |
| Selective reporting (reporting bias) | Low risk | All outcomes had been reported. |
| Other bias | Low risk | No other sources of bias. |

**Wang YC 2021**

| **Bias** | **Authors' judgement** | **Support for judgement** |
| --- | --- | --- |
| Random sequence generation (selection bias) | Low risk | SPSS 22.0 statistical software was used to generate a random number table. |
| Allocation concealment (selection bias) | Unclear risk | Not mentioned. |
| Blinding of participants and personnel (performance bias) | Unclear risk | Not mentioned. |
| Blinding of outcome assessment (detection bias) | Unclear risk | Not mentioned. |
| Incomplete outcome data (attrition bias) | Low risk | No missing data. |
| Selective reporting (reporting bias) | Low risk | All outcomes had been reported. |
| Other bias | Low risk | No other sources of bias. |

**Wei JF 2021**

| **Bias** | **Authors' judgement** | **Support for judgement** |
| --- | --- | --- |
| Random sequence generation (selection bias) | Low risk | A random number table was applied. |
| Allocation concealment (selection bias) | Unclear risk | Not mentioned. |
| Blinding of participants and personnel (performance bias) | Unclear risk | Not mentioned. |
| Blinding of outcome assessment (detection bias) | Unclear risk | Not mentioned. |
| Incomplete outcome data (attrition bias) | Low risk | No missing data. |
| Selective reporting (reporting bias) | Low risk | All outcomes had been reported. |
| Other bias | Low risk | No other sources of bias. |

**Xie Y 2021**

| **Bias** | **Authors' judgement** | **Support for judgement** |
| --- | --- | --- |
| Random sequence generation (selection bias) | Low risk | A random number table was applied. |
| Allocation concealment (selection bias) | Unclear risk | Not mentioned. |
| Blinding of participants and personnel (performance bias) | Unclear risk | Not mentioned. |
| Blinding of outcome assessment (detection bias) | Unclear risk | Not mentioned. |
| Incomplete outcome data (attrition bias) | Low risk | No missing data. |
| Selective reporting (reporting bias) | Low risk | All outcomes had been reported. |
| Other bias | Low risk | No other sources of bias. |

**Xu ZQ 2015**

| **Bias** | **Authors' judgement** | **Support for judgement** |
| --- | --- | --- |
| Random sequence generation (selection bias) | Unclear risk | The specific method of randomization which was chosen was not reported. |
| Allocation concealment (selection bias) | Unclear risk | Not mentioned. |
| Blinding of participants and personnel (performance bias) | Unclear risk | Not mentioned. |
| Blinding of outcome assessment (detection bias) | Unclear risk | Not mentioned. |
| Incomplete outcome data (attrition bias) | Low risk | No missing data. |
| Selective reporting (reporting bias) | Low risk | All outcomes had been reported. |
| Other bias | Low risk | No other sources of bias. |

**Zhang X 2020**

| **Bias** | **Authors' judgement** | **Support for judgement** |
| --- | --- | --- |
| Random sequence generation (selection bias) | Low risk | A random number table was applied. |
| Allocation concealment (selection bias) | Unclear risk | Not mentioned. |
| Blinding of participants and personnel (performance bias) | Unclear risk | Not mentioned. |
| Blinding of outcome assessment (detection bias) | Unclear risk | Not mentioned. |
| Incomplete outcome data (attrition bias) | Low risk | No missing data. |
| Selective reporting (reporting bias) | Low risk | All outcomes had been reported. |
| Other bias | Low risk | No other sources of bias. |

**Zhao H 2015**

| **Bias** | **Authors' judgement** | **Support for judgement** |
| --- | --- | --- |
| Random sequence generation (selection bias) | Low risk | SPSS 22.0 statistical software was used to generate a random number table. |
| Allocation concealment (selection bias) | Low risk | An independent physician who did not participate in the treatment and efficacy evaluation was permitted to disclose the envelopes according to the number sequence, and the patients were grouped based on the enclosed allocation plan. |
| Blinding of participants and personnel (performance bias) | Unclear risk | Not mentioned. |
| Blinding of outcome assessment (detection bias) | Unclear risk | Not mentioned. |
| Incomplete outcome data (attrition bias) | Low risk | No missing data. |
| Selective reporting (reporting bias) | Low risk | All outcomes had been reported. |
| Other bias | Low risk | No other sources of bias. |

**Zhao HB 2022**

| **Bias** | **Authors' judgement** | **Support for judgement** |
| --- | --- | --- |
| Random sequence generation (selection bias) | Low risk | A random number table was applied. |
| Allocation concealment (selection bias) | Unclear risk | Not mentioned. |
| Blinding of participants and personnel (performance bias) | Unclear risk | Not mentioned. |
| Blinding of outcome assessment (detection bias) | Unclear risk | Not mentioned. |
| Incomplete outcome data (attrition bias) | Low risk | No missing data. |
| Selective reporting (reporting bias) | Low risk | All outcomes had been reported. |
| Other bias | Low risk | No other sources of bias. |

**Supplementary Material Table 4. Sensitivity analyses for outcomes.**

**Visual Analog Scale (VAS)**

| **Excluded study** | **Intervention**  **group(n)** | **Control**  **group(n)** | **MD (95% CI)** | **P value** | **Heterogeneity**  **test** | **Effect model** |
| --- | --- | --- | --- | --- | --- | --- |
| Before excluding | 527 | 528 | -1.68 [-2.08, -1.28] | <0.00001 | Tau^2^=0.38 I^2^=89% | Random |
| Gu WY 2019 | 486 | 487 | -1.66 [-2.08, -1.23] | <0.00001 | Tau^2^=0.40 I^2^=90% | Random |
| Qiu CM 2011 | 467 | 468 | -1.67 [-2.10, -1.24] | <0.00001 | Tau^2^=0.40 I^2^=90% | Random |
| Xie Y 2021 | 474 | 475 | -1.68 [-2.13, -1.23] | <0.00001 | Tau^2^=0.44 I^2^=90% | Random |
| Zhao H 2015 | 465 | 466 | -1.60 [-2.00, -1.19] | <0.00001 | Tau^2^=0.35 I^2^=88% | Random |
| Du HX 2012 | 489 | 490 | -1.70 [-2.12, -1.27] | <0.00001 | Tau^2^=0.41 I^2^=90% | Random |
| Hu N 2019 | 484 | 485 | -1.68 [-2.12, -1.24] | <0.00001 | Tau^2^=0.43 I^2^=90% | Random |
| Chen L 2016 | 487 | 488 | -1.63 [-2.04, -1.21] | <0.00001 | Tau^2^=0.38 I^2^=89% | Random |
| Zhang X 2020 | 467 | 468 | -1.63 [-2.04, -1.21] | <0.00001 | Tau^2^=0.37 I^2^=87% | Random |
| Gao LA 2023 | 479 | 479 | -1.70 [-2.12, -1.28] | <0.00001 | Tau^2^=0.40 I^2^=90% | Random |
| Zhao HB 2022 | 497 | 498 | -1.82 [-2.13, -1.52] | <0.00001 | Tau^2^=0.16 I^2^=75% | Random |
| Xu ZQ 2015 | 475 | 476 | -1.73 [-2.20, -1.26] | <0.00001 | Tau^2^=0.49 I^2^=89% | Random |

**Fugl-Meyer Assessment of the Upper Extremity**

| **Excluded study** | **Intervention**  **group(n)** | **Control**  **group(n)** | **MD (95% CI)** | **P value** | **Heterogeneity**  **test** | **Effect model** |
| --- | --- | --- | --- | --- | --- | --- |
| Before excluding | 1044 | 1034 | 8.76 [7.00, 10.53] | <0.00001 | Tau^2^=16.4 I^2^=94% | Random |
| Han B 2010 | 979 | 971 | 8.84 [6.96, 10.72] | <0.00001 | Tau^2^=17.92 I^2^=94% | Random |
| Huang Z 2021 | 1000 | 990 | 8.82 [7.01, 10.63] | <0.00001 | Tau^2^=16.61 I^2^=94% | Random |
| Liu CY 2017 | 1014 | 1004 | 8.56 [6.75, 10.36] | <0.00001 | Tau^2^=16.47 I^2^=94% | Random |
| Qiu CM 2011 | 984 | 974 | 8.44 [6.71, 10.17] | <0.00001 | Tau^2^=14.84 I^2^=93% | Random |
| Wang RH 2017 | 988 | 979 | 8.86 [7.05, 10.67] | <0.00001 | Tau^2^=16.59 I^2^=94% | Random |
| Wei JF 2021 | 993 | 983 | 8.76 [6.87, 10.66] | <0.00001 | Tau^2^=18.20 I^2^=94% | Random |
| Xie Y 2021 | 991 | 981 | 8.72 [6.87, 10.57] | <0.00001 | Tau^2^=17.72 I^2^=94% | Random |
| Chen SQ 2023 | 1099 | 999 | 8.46 [6.67, 10.25] | <0.00001 | Tau^2^=16.04 I^2^=94% | Random |
| Huang ZX 2022 | 1014 | 1004 | 8.52 [6.81, 10.24] | <0.00001 | Tau^2^=14.52 I^2^=91% | Random |
| Sun J 2022 | 1016 | 1006 | 8.78 [6.99, 10.57] | <0.00001 | Tau^2^=16.5 I^2^=94% | Random |
| Wang L 2023 | 1014 | 1004 | 8.94 [7.16, 10.72] | <0.00001 | Tau^2^=16.30 I^2^=94% | Random |
| Zhao HB 2022 | 1014 | 1004 | 8.82 [7.01, 10.62] | <0.00001 | Tau^2^=16.55 I^2^=94% | Random |
| Chen L 2016 | 1004 | 994 | 8.72 [6.92, 10.53] | <0.00001 | Tau^2^=16.58 I^2^=94% | Random |
| Huang ZZ 2017 | 988 | 986 | 8.80 [6.95, 10.65] | <0.00001 | Tau^2^=17.31 I^2^=94% | Random |
| Jin HM 2013 | 1014 | 1004 | 8.70 [6.88, 10.51] | <0.00001 | Tau^2^=16.70 I^2^=94% | Random |
| Liu CH 2020 | 1004 | 994 | 9.07 [7.30, 10.84] | <0.00001 | Tau^2^=15.71 I^2^=94% | Random |
| Ding CM 2022 | 999 | 989 | 8.66 [6.83, 10.49] | <0.00001 | Tau^2^=16.91 I^2^=94% | Random |
| Tong QS 2023 | 1014 | 1005 | 8.93 [7.13, 10.73] | <0.00001 | Tau^2^=16.36 I^2^=94% | Random |
| Wang YC 2021 | 994 | 984 | 9.01 [7.26, 10.75] | <0.00001 | Tau^2^=15.12 I^2^=93% | Random |
| Du HX 2012 | 1006 | 996 | 9.10 [7.41, 10.79] | <0.00001 | Tau^2^=13.99 I^2^=93% | Random |
| Hu N 2019 | 1001 | 991 | 8.80 [6.95, 10.65] | <0.00001 | Tau^2^=17.25 I^2^=94% | Random |
| He QC 2013 | 984 | 973 | 8.48 [6.68, 10.47] | <0.00001 | Tau^2^=16.15 I^2^=94% | Random |
| Xu ZQ 2015 | 992 | 982 | 8.79 [6.96, 10.21] | <0.00001 | Tau^2^=16.82 I^2^=94% | Random |
| Gao LA 2023 | 996 | 985 | 8.68 [6.88, 10.47] | <0.00001 | Tau^2^=16.51 I^2^=94% | Random |

**Modified Barthel index**

| **Excluded study** | **Intervention**  **group(n)** | **Control**  **group(n)** | **MD (95% CI)** | **P value** | **Heterogeneity**  **test** | **Effect model** |
| --- | --- | --- | --- | --- | --- | --- |
| Before excluding | 357 | 357 | 10.27 [6.16, 14.37] | <0.00001 | Tau^2^=28.91 I^2^=81% | Random |
| Gu WY 2019 | 316 | 316 | 9.23 [5.06, 13.38] | <0.00001 | Tau^2^=26.38 I^2^=81% | Random |
| Qiu CM 2011 | 297 | 297 | 8.59 [5.61, 11.57] | =0.05 | Tau^2^=8.48 I^2^=51% | Random |
| Shen LH 2013 | 317 | 317 | 10.66 [5.89, 15.43] | <0.00001 | Tau^2^=35.44 I^2^=82% | Random |
| Sun J 2022 | 329 | 329 | 9.94 [5.59, 10.17] | <0.00001 | Tau^2^=30.40 I^2^=83% | Random |
| Wang L 2023 | 327 | 327 | 11.25 [7.13, 15.37] | <0.00001 | Tau^2^=25.88 I^2^=80% | Random |
| Gao LA 2023 | 309 | 308 | 10.27 [5.82, 14.71] | <0.00001 | Tau^2^=31.34 I^2^=83% | Random |
| Niu L 2022 | 317 | 317 | 10.49 [5.64, 15.34] | <0.00001 | Tau^2^=37.06 I^2^=83% | Random |
| Tong QS 2023 | 327 | 328 | 10.93 [6.47, 15.39] | <0.00001 | Tau^2^=30.18 I^2^=81% | Random |
| Liu CH 2020 | 317 | 317 | 10.71 [6.12, 15.29] | <0.00001 | Tau^2^=32.49 I^2^=82% | Random |

**Barthel index**

| **Excluded study** | **Intervention**  **group(n)** | **Control**  **group(n)** | **MD (95% CI)** | **P value** | **Heterogeneity**  **test** | **Effect model** |
| --- | --- | --- | --- | --- | --- | --- |
| Before excluding | 357 | 357 | 10.27 [6.16, 14.37] | <0.00001 | Tau^2^=28.91 I^2^=81% | Random |
| Gu WY 2019 | 316 | 316 | 9.23 [5.06, 13.38] | <0.00001 | Tau^2^=26.38 I^2^=81% | Random |
| Qiu CM 2011 | 297 | 297 | 8.59 [5.61, 11.57] | =0.05 | Tau^2^=8.48 I^2^=51% | Random |
| Shen LH 2013 | 317 | 317 | 10.66 [5.89, 15.43] | <0.00001 | Tau^2^=35.44 I^2^=82% | Random |
| Sun J 2022 | 329 | 329 | 9.94 [5.59, 10.17] | <0.00001 | Tau^2^=30.40 I^2^=83% | Random |
| Wang L 2023 | 327 | 327 | 11.25 [7.13, 15.37] | <0.00001 | Tau^2^=25.88 I^2^=80% | Random |
| Gao LA 2023 | 309 | 308 | 10.27 [5.82, 14.71] | <0.00001 | Tau^2^=31.34 I^2^=83% | Random |
| Niu L 2022 | 317 | 317 | 10.49 [5.64, 15.34] | <0.00001 | Tau^2^=37.06 I^2^=83% | Random |
| Tong QS 2023 | 327 | 328 | 10.93 [6.47, 15.39] | <0.00001 | Tau^2^=30.18 I^2^=81% | Random |


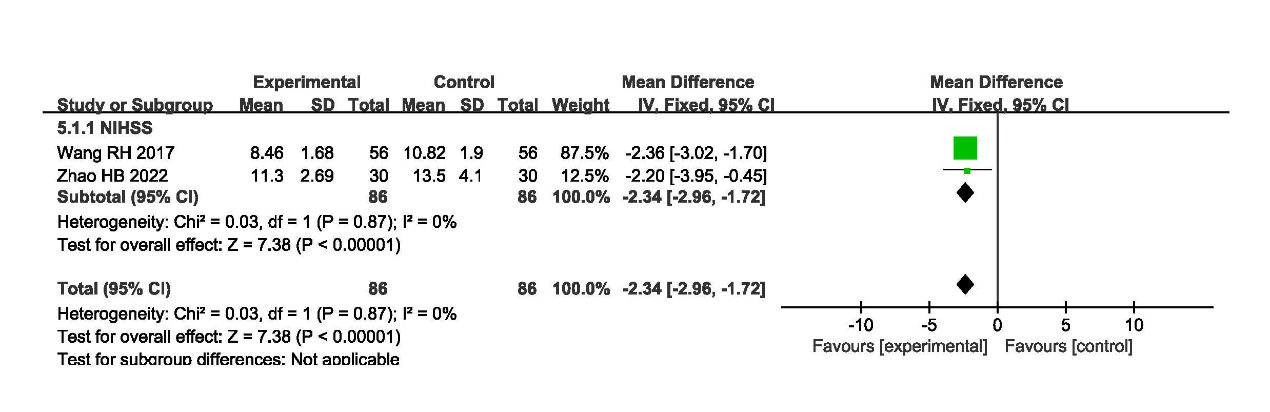


**Supplementary Material Figure 1. Forest plot of NIHSS.**

**
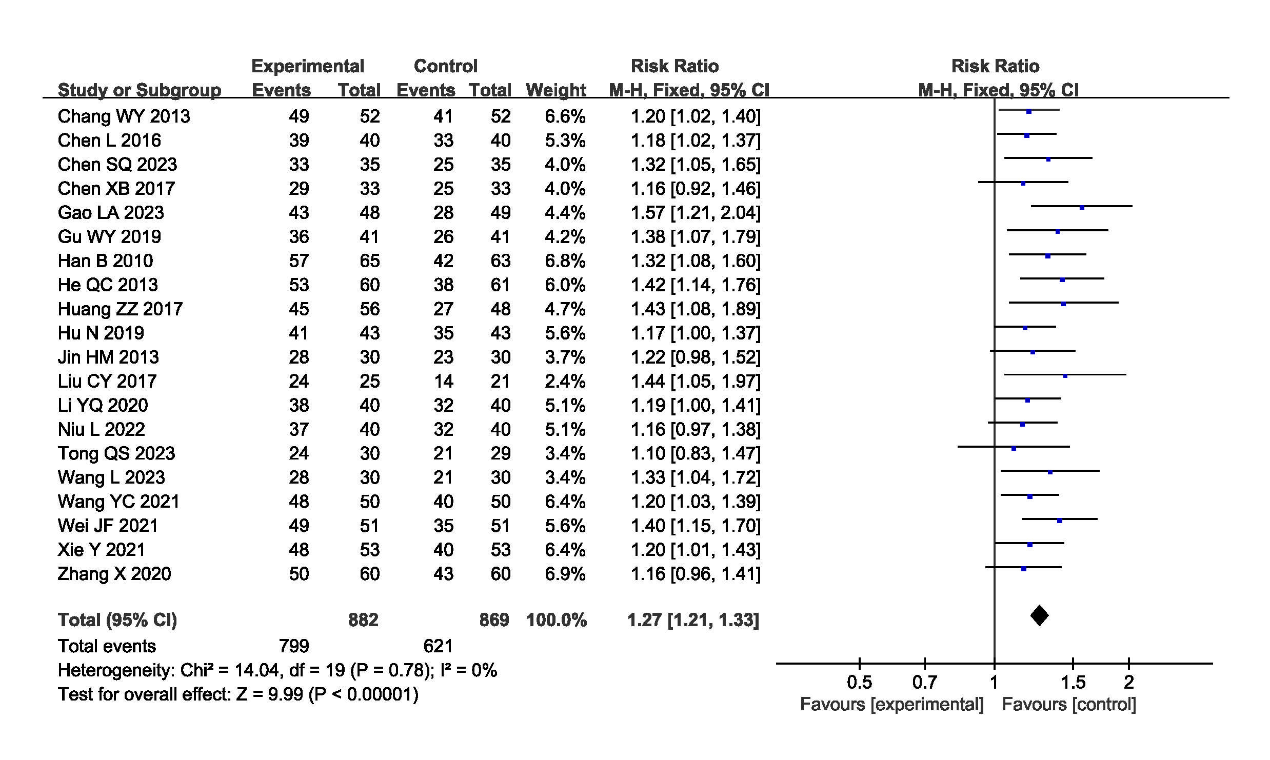
**

**Supplementary Material Figure 2. Forest plot of TERs.**

**
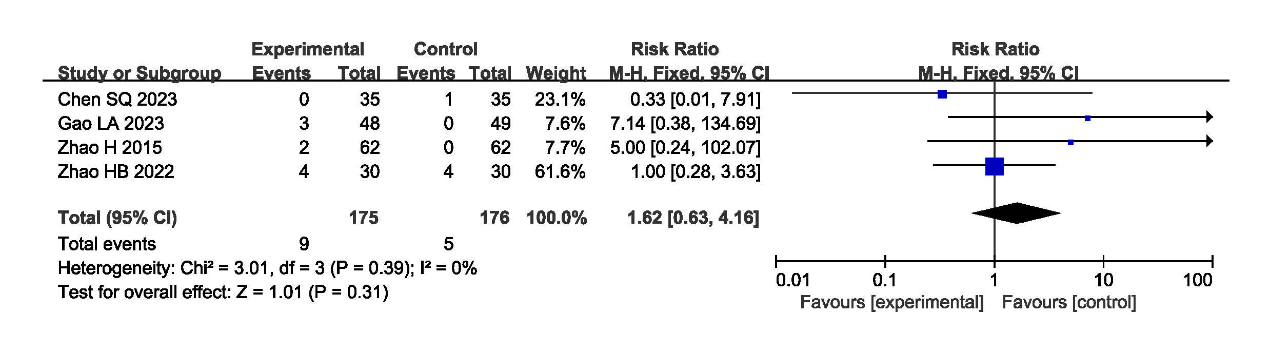
**

**Supplementary Material Figure 3. Forest plot of AEs.**
